# Cyclic Diguanylate Regulates Virulence Factor Genes via Multiple Riboswitches in *Clostridium difficile*

**DOI:** 10.1128/mSphere.00423-18

**Published:** 2018-10-24

**Authors:** Robert W. McKee, Carissa K. Harvest, Rita Tamayo

**Affiliations:** a Department of Microbiology and Immunology, University of North Carolina at Chapel Hill School of Medicine, Chapel Hill, North Carolina, USA; University of Iowa

**Keywords:** *Clostridium difficile*, biofilms, c-di-GMP, cyclic diguanylate, flagellar motility, riboswitch

## Abstract

The intracellular signaling molecule cyclic diguanylate (c-di-GMP) regulates many processes in bacteria, with a central role in controlling the switch between motile and nonmotile lifestyles. Recent work has shown that in Clostridium difficile (also called Clostridioides difficile), c-di-GMP regulates swimming and surface motility, biofilm formation, toxin production, and intestinal colonization. In this study, we determined the transcriptional regulon of c-di-GMP in C. difficile, employing overexpression of a diguanylate cyclase gene to artificially manipulate intracellular c-di-GMP. Consistent with prior work, c-di-GMP regulated the expression of genes involved in swimming and surface motility. c-di-GMP also affected the expression of multiple genes encoding cell envelope proteins, several of which affected biofilm formation *in vitro*. A substantial proportion of the c-di-GMP regulon appears to be controlled either directly or indirectly via riboswitches. We confirmed the functionality of 11 c-di-GMP riboswitches, demonstrating their effects on downstream gene expression independent of the upstream promoters. The class I riboswitches uniformly functioned as “off” switches in response to c-di-GMP, while class II riboswitches acted as “on” switches. Transcriptional analyses of genes 3′ of c-di-GMP riboswitches over a broad range of c-di-GMP levels showed that relatively modest changes in c-di-GMP levels are capable of altering gene transcription, with concomitant effects on microbial behavior. This work expands the known c-di-GMP signaling network in C. difficile and emphasizes the role of the riboswitches in controlling known and putative virulence factors in C. difficile.

**IMPORTANCE** In Clostridium difficile, the signaling molecule c-di-GMP regulates multiple processes affecting its ability to cause disease, including swimming and surface motility, biofilm formation, toxin production, and intestinal colonization. In this study, we used RNA-seq to define the transcriptional regulon of c-di-GMP in C. difficile. Many new targets of c-di-GMP regulation were identified, including multiple putative colonization factors. Transcriptional analyses revealed a prominent role for riboswitches in c-di-GMP signaling. Only a subset of the 16 previously predicted c-di-GMP riboswitches were functional *in vivo* and displayed potential variability in their response kinetics to c-di-GMP. This work underscores the importance of studying c-di-GMP riboswitches in a relevant biological context and highlights the role of the riboswitches in controlling gene expression in C. difficile.

## Abstract (precis)

In Clostridium difficile, the signaling molecule c-di-GMP regulates multiple processes affecting its ability to cause disease, including swimming and surface motility, biofilm formation, toxin production, and intestinal colonization. In this study, we used RNA-seq to define the transcriptional regulon of c-di-GMP in C. difficile. Many new targets of c-di-GMP regulation were identified, including multiple putative colonization factors. Transcriptional analyses revealed a prominent role for riboswitches in c-di-GMP signaling. Only a subset of the 16 previously predicted c-di-GMP riboswitches were functional *in vivo* and displayed potential variability in their response kinetics to c-di-GMP. This work underscores the importance of studying c-di-GMP riboswitches in a relevant biological context and highlights the role of the riboswitches in controlling gene expression in C. difficile.

## INTRODUCTION

Cyclic diguanylate (c-di-GMP) is nearly ubiquitous in bacteria and well known to regulate a variety of processes, particularly the switch between motile and sessile states (1–4). In some pathogens, c-di-GMP also controls virulence factor production (5–10). In response to largely undefined extracellular cues, the intracellular c-di-GMP concentration is modulated through the opposing activities of diguanylate cyclases (DGCs) and phosphodiesterases (PDEs). These enzymes may be controlled at the transcriptional and posttranslational level to adjust intracellular c-di-GMP. Many bacteria encode multiple DGCs and PDEs leading to complex c-di-GMP signaling networks. Changes in intracellular c-di-GMP are sensed by specific receptors that respond with changes to their activity or function. Multiple protein receptors of c-di-GMP receptors have been identified, including PilZ domains, diguanylate cyclases containing I-sites, GIL proteins, MshEN domains, and the Cle subfamily of CheY proteins (11–16). c-di-GMP can also bind to certain transcription factors and alter their activity (17–19). In addition to these protein receptors, c-di-GMP can bind to two distinct RNA structures, the class I (GEMM) and class II c-di-GMP riboswitches, to carry out its regulatory function (20, 21). Riboswitches are found in the 5′ untranslated region (UTR) of some transcripts. Binding of a ligand to the riboswitch alters the secondary structure of the RNA to promote or inhibit transcript termination, mRNA stability, or translation initiation (22, 23). Characterization of gene regulation by c-di-GMP riboswitches has largely relied on experiments using purified c-di-GMP and transcripts generated through *in vitro* transcription or using heterologous hosts (20, 25). However, little work has been done to directly test the role of c-di-GMP riboswitches in their native genetic contexts *in vivo*.

Clostridium difficile is an obligate anaerobe capable of causing intestinal disease ranging from mild diarrhea to potentially fatal pseudomembranous colitis and toxic megacolon. Recent work suggests that c-di-GMP plays a key role in controlling the production of surface structures involved in host colonization by C. difficile. For example, C. difficile produces peritrichous flagella that are involved in intestinal colonization, and the flagellum itself may serve as an adhesin (26–28). The expression of flagellar genes, flagellum biosynthesis, and motility are negatively regulated by c-di-GMP (29). C. difficile also produces type IV pili (TFP) that participate in autoaggregation, biofilm formation, adherence to epithelial cells, and persistence of C. difficile in the mammalian intestine (30–32). The production of TFP is positively regulated by c-di-GMP, also at the level of transcriptional regulation (30). In addition, two sortase-dependent surface proteins, encoded by CD630_28310 and CD630_32460, are positively regulated by c-di-GMP (21, 33), although the contribution of these proteins to adherence and host colonization has not been reported. Interestingly, the ZmpI zinc-dependent metalloprotease, which cleaves the CD630_28310 and CD630_32460 proteins, is negatively regulated by c-di-GMP (33).

C. difficile strain 630 encodes 37 proteins with putative or demonstrated DGC or PDE activity (25, 29, 34). Additionally, C. difficile 630 encodes 16 predicted class I and class II c-di-GMP sensing riboswitches, more than any sequenced bacterial genome outside a few deltaproteobacteria, suggesting an important role for c-di-GMP signaling through riboswitches in this organism (2, 20, 21). The presence of truncated RNAs corresponding to 7 of the c-di-GMP riboswitches has been confirmed by Northern blotting (35). Notably, each of the loci described above, CD630_28310, CD630_32460, *zmpI*, and *pilA1* (TFP major pilin gene), and the *flgB* operon (flagellar genes) are preceded by a c-di-GMP riboswitch (20, 21). Regulation of *pilA1* expression has been demonstrated to occur through the Cdi-2-4 riboswitch in the 5′ UTR of the transcript (30–32). In the presence of c-di-GMP, the Cdi-2-4 riboswitch assumes a secondary structure that allows transcription read-through and expression of the downstream TFP genes, consistent with the observed positive regulation of pilus gene expression and TFP production by c-di-GMP *in vivo* (30, 31). Conversely, c-di-GMP binding to the Cdi-1-3 riboswitch in the 5′ UTR of the *flgB* operon mRNA causes transcript termination, leading to a decrease in the transcription of *flgB* and other genes in the early-stage flagellar operon (20, 29, 35).

The processes regulated by c-di-GMP in C. difficile identified to date have been identified based on the presence of putative c-di-GMP riboswitches upstream of the relevant loci, and studies have specifically focused on those loci affecting measurable phenotypes. Yet evidence suggests that additional c-di-GMP-regulated factors remain to be identified. For example, the adherence behaviors that occur in C. difficile in response to elevated c-di-GMP can be attributed only in part to regulation of TFP and flagellar biosynthesis (29–32). Inactivation of TFP genes reduces, but does not eliminate, biofilm formation in response to c-di-GMP in C. difficile 630Δerm (31). Similarly, c-di-GMP-mediated increases in adherence to epithelial cells *in vitro* can be only partly explained by inhibition of flagellum biosynthesis (27, 32). The broader role of c-di-GMP signaling in C. difficile has not been determined.

In this study, we used RNA-seq to identify additional factors regulated by c-di-GMP in C. difficile. The analysis took advantage of a previously developed strategy for artificial manipulation of c-di-GMP in C. difficile, which allowed us to compare the transcriptomes of C. difficile strain 630Δerm with elevated intracellular c-di-GMP concentrations and basal c-di-GMP levels. Transcriptional analyses demonstrated a prominent role for riboswitches in the c-di-GMP signaling system of C. difficile and allowed us to examine the *in vivo* functionality of the 16 predicted c-di-GMP riboswitches in this organism. Eleven showed an ability to regulate gene expression in response to c-di-GMP, with class I riboswitches functioning as “off” switches and class II riboswitches functioning as “on” switches. Differences in responsiveness to changes in c-di-GMP were observed, suggesting that individual riboswitches of the same class display distinct thresholds of activation. This work underscores the importance of examining c-di-GMP riboswitch function in a relevant biological context and highlights the role of the riboswitches in controlling known and putative virulence factors in C. difficile.

## RESULTS

### c-di-GMP controls the transcription of a large number of genes in C. difficile.

**FIG 1:**
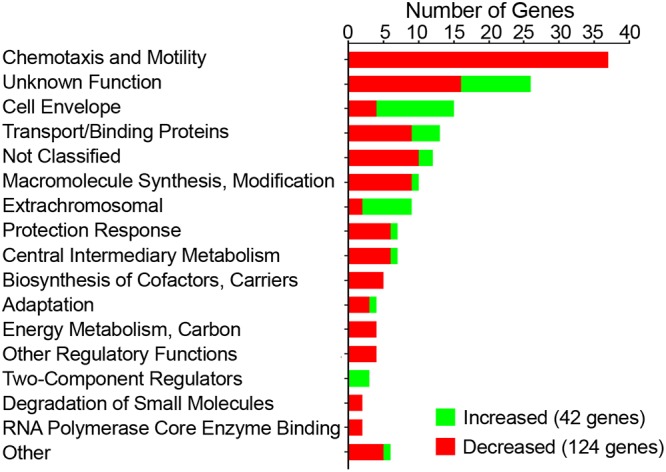
C. difficile genes regulated by c-di-GMP grouped by Riley classification of predicted gene products. Genes were included if the fold change in expression (differences in RPKM) was greater than 2-fold and *P* value was <0.05 after Bonferroni’s correction for multiple comparisons.

To identify the genes regulated by c-di-GMP, we used RNA-seq to compare the transcriptomes of C. difficile 630Δerm with wild-type or elevated c-di-GMP levels. C. difficile with pMC-Pcpr (vector control) served as the wild type, which we previously determined to contain low levels of c-di-GMP (near the limit of detection by UPLC-MS) (29). To increase intracellular c-di-GMP, the diguanylate cyclase gene *dccA* was expressed from a plasmid, pDccA, under the control of the nisin-inducible *cpr* promoter (29). C. difficile 630Δerm bearing pDccA or vector control was grown to mid-exponential phase with 1 μg/ml nisin, and RNA was processed for sequencing. Genes were considered to be regulated using the following criteria: changes in reads mapped per kilobase per million reads (RPKM) of >2-fold between the two conditions and *P* < 0.05 by Bonferroni’s correction. A complete list of the differentially expressed genes is found in Table S3 in the supplemental material. These genes were grouped according to the predicted Riley functional class of their encoded proteins (Fig. 1) (36, 37).

A total of 166 genes met the criteria, with 124 genes negatively regulated and 42 positively regulated by c-di-GMP (Fig. 1). The largest class of genes regulated by c-di-GMP were genes involved in chemotaxis and flagellar motility (37 total genes). These genes account for nearly 25% of the c-di-GMP-regulated genes identified. Consistent with previous work showing that c-di-GMP negatively regulates flagellar motility, all these genes showed decreased expression in response to increased c-di-GMP (pDccA strain) (Fig. 1). The next largest set of c-di-GMP-regulated genes are known or predicted to encode proteins that localize to the cell envelope. Among this set are genes involved in biosynthesis of TFP, which showed increased expression in response to c-di-GMP, consistent with previous studies (30, 32). Three genes encoding putative surface proteins, CD630_27970, CD630_28310, and CD630_32460, were also in this category, and all three genes are preceded by predicted c-di-GMP riboswitches. The third major class of c-di-GMP-regulated genes encode proteins predicted to be involved in transport/binding. Of these, seven are predicted PTS system proteins that are likely involved in importing sugars or sugar alcohols, and six are predicted components of ABC-type transporters. Across the remaining classes, several of the remaining genes are predicted to encode phage proteins, proteins involved in the transfer of mobile genetic elements, and proteins involved in iron regulation (e.g., FeoB1) or oxidative stress (e.g., Rbr1 and TrxA1).

### Increased intracellular c-di-GMP moderately affects transcription of c-di-GMP metabolism genes.

Because ectopic expression of *dccA* greatly increases intracellular c-di-GMP, we considered that the C. difficile c-di-GMP signaling system might compensate by altering other c-di-GMP enzymes to buffer against altered c-di-GMP. To address this possibility, we examined the RNA-seq data for genes encoding the GGDEF and/or EAL domains that synthesize and degrade c-di-GMP, respectively. As expected, transcription of *dccA* was significantly elevated (8.6-fold) in C. difficile pDccA (Fig. S1). One other locus, CD630_07570 encoding a GGDEF-EAL domain phosphodiesterase (25, 34), showed a significant (5.1-fold) decrease in transcription, which suggests a further increase in intracellular c-di-GMP. Several other c-di-GMP metabolism genes showed modest changes in expression but did not meet the defined statistical cutoff. As a group, those containing GGDEF domains, most of which are confirmed DGCs (25, 34), showed no particular upward or downward trend in transcription in C. difficile pDccA. However, those with tandem GGDEF-EAL domains, most of which function as PDEs (25, 34), showed a trend toward increased transcription. When analyzed individually by *t* test, many of the genes showed statistically significant changes in expression. These results suggest that C. difficile may cumulatively buffer against increased c-di-GMP by moderately increasing the expression of multiple phosphodiesterase genes. It also remains possible that existing DGC and PDE enzymes are regulated posttranslationally in response to elevated c-di-GMP.

10.1128/mSphere.00423-18.1 FIG S1 The effect of artificially increased c-di-GMP on GGDEF and EAL domain-encoding genes. Shown are the means and standard deviations of reads normalized per kilobase per million reads (RPKM) for genes encoding GGDEF and/or EAL domain proteins. #, at least 2-fold change and *P* < 0.05 after Bonferroni’s correction; *, *P* < 0.05 by Student’s *t* test comparing RPKM values in C. difficile with vector versus pDccA. Download FIG S1, PDF file, 0.3 MB. Copyright © 2018 McKee et al. 2018 McKee et al. This content is distributed under the terms of the Creative Commons Attribution 4.0 International license.

### Genes predicted to be controlled by class I and class II riboswitches are well represented among c-di-GMP-regulated genes.

A total of 16 c-di-GMP riboswitches are predicted in C. difficile 630: twelve class I (GEMM) riboswitches and four class II riboswitches (20, 21). Of the 12 class I riboswitches, transcripts corresponding to the genes downstream of 7 were significantly altered (Table 1). Transcript abundances for six of these loci were significantly lower in C. difficile pDccA than the vector control, with fold changes ranging from −4.66 to −62.61. These results suggest that these class I riboswitches generally function as “off” switches in response to c-di-GMP. The negatively regulated loci encode flagella (Cdi-1-3), a calcium binding putative adhesin (Cdi-1-2), and the zinc-dependent metalloprotease ZmpI (Cdi-1-12) (38). The remaining three encode hypothetical proteins (Cdi-1-8, Cdi-1-9, and Cdi-1-11). In contrast, the expression of CD630_19900, the gene downstream of Cdi-1-1, was increased more than 30-fold, and this gene is the sole class I riboswitch-regulated gene positively regulated by c-di-GMP.

**TABLE 1:** Changes in transcript abundance for c-di-GMP riboswitches and the downstream genes

Riboswitch^*a*^	Chromosome region start^*a*^^,^^*b*^	Fold change RS (pDccA/vector)^*c*^	Downstream gene	Chromosome region^*b*^	Fold change gene (pDccA/vector)^*d*^
Cdi-1-1	(−) 2296134	**13.44**^*f*^	CD630_19900	(−) 2295867...2296352	**31.90**
Cdi-1-2	(−) 3266578	*−15.63*^*f*^	CD630_27970	(−) 3260792...3266755	*−7.15*
Cdi-1-3	(+) 308778	*−2.74*	CD630_02450 *(flgB*)	(+) 309272...309589	*−15.06*
Cdi-1-4	(+) 3379981	1.02	ND^*e*^		
Cdi-1-5	(−) 1142269	1.03	ND		
Cdi-1-6	(+) 2285923	*−6.41*	ND		
Cdi-1-7	(+) 2907226	1.19	ND		
Cdi-1-8	(+) 2297492	*−9.25*	CD630_19903	(+) 2297643...2297819	*−7.78*
Cdi-1-9	(+) 2671809	*−4.81*	CD630_23090	(+) 2671951...2672127	*−4.66*
Cdi-1-10	(−) 1653520	*−2.07*	ND		
Cdi-1-11	(+) 3936240	*−8.49*	CD630_33682	(+) 3936389...3936565	*−8.90*
Cdi-1-12	(−) 3303074	*−31.30*	CD630_28300 (*zmpI*)	(−) 3302613...3303275	*−62.61*
Cdi-2-1	(−) 3801063	**4.48**	CD630_32460	(−) 3798299...3800482	**4.29**
Cdi-2-2	(−) 3826609	**2.34**	CD630_32670	(−) 3825352...3826029	**18.78**
Cdi-2-3	(−) 3306681	1.10	CD630_28310	(−) 3303646...3306564	**42.51**
Cdi-2-4	(−) 4105796	**4.10**	CD630_35130 (*pilA1*)	(−) 4105120...4105635	**11.74**

a Riboswitch naming and start sites based on predictions by Sudarsan et al. (20) and Lee et al. (21).

b (+/−) indicates sense versus antisense strand.

c Fold change for the riboswitch region only.

d Fold change for the gene 3′ of the riboswitch.

e ND, no downstream genes or transcripts detected.

f Boldface and italic indicate significantly increased and decreased abundance relative to the vector control condition, respectively.

For the remaining five class I riboswitches (Cdi-1-4, Cdi-1-5, Cdi-1-6, Cdi-1-7, and Cdi-1-10), no coding sequence was detectable downstream of the riboswitch based on either the genome annotation or analysis of the reads in the regions (Table 1 and Fig. S2). Transcript reads corresponding to the riboswitch sequences themselves were detected (Fig. S2), albeit at very low levels. Transcripts for annotated genes overlapping riboswitches Cdi-1-6 and Cdi-1-10 significantly differed between C. difficile pDccA and the control. These results suggest these mRNAs are nonfunctional riboswitches or have functions other than gene regulation in *cis*.

10.1128/mSphere.00423-18.2 FIG S2 Evidence of transcription corresponding to the class I riboswitches lacking a downstream open reading frame. Reads in red correspond to the sense strand; reads in green, to the antisense strand. Reads in yellow map to more than one location of the genome. Top panels of each image show reads from C. difficile 630 pDccA, and bottom panels show reads from the vector control. The genome positions are indicated at the top of each image. The annotated riboswitch is shown in blue block arrows, as are genes in the region. The number of reads can be estimated using the logarithmic scale on the left. Download FIG S2, PDF file, 0.6 MB. Copyright © 2018 McKee et al. 2018 McKee et al. This content is distributed under the terms of the Creative Commons Attribution 4.0 International license.

The class I c-di-GMP riboswitches are generally highly conserved in C. difficile, with Cdi-1-1, Cdi-1-6, Cdi-1-8, Cdi-1-9, Cdi-1-10, Cdi-1-11, and Cdi-1-12 present in all 54 complete genomes available on NCBI (Fig. S3). Cdi-1-2 and Cdi-1-3 are absent in a subset of strains that also lack the downstream CD630_27970 and *flgB* genes, respectively. Notably, the nonfunctional Cdi-1-4 and Cdi-1-5 and their downstream regions appear to be duplications in C. difficile 630 and 630Δerm, and they are absent from most available C. difficile genomes (Fig. S3). Cdi-1-4 and Cdi-1-5 are most similar to Cdi-1-7, which may explain their common lack of function.

10.1128/mSphere.00423-18.3 FIG S3 Conservation of riboswitch regions across C. difficile genomes. The conservation of c-di-GMP riboswitch sequences in the C. difficile complete genomes available through NCBI was determined using BLASTn with query sequences from C. difficile 630. Because of high sequence identity among the aptamers and some sequence duplications, ∼250 nucleotides downstream of the riboswitches were included to distinguish between them. Conservation of select c-di-GMP riboswitch-controlled genes, CD630_02450 (*flgB*), CD630_32460, and CD630_32670, was also examined; their respective riboswitches are noted above the locus numbers. Black squares indicate the presence of the sequence (greater than or equal to 90% identity and >97% coverage). Gray squares indicate overall conservation, but with reduced identity (80% to 90% identity and >97% coverage). White squares indicate the absence of the riboswitch. Download FIG S3, PDF file, 0.3 MB. Copyright © 2018 McKee et al. 2018 McKee et al. This content is distributed under the terms of the Creative Commons Attribution 4.0 International license.

In contrast to the majority of class I riboswitch loci, the transcript abundances for the four genes downstream of class II riboswitches were significantly higher (4.29- to 42.51-fold) in C. difficile pDccA than in the vector control, indicating that class II riboswitches are uniformly “on” switches in C. difficile. Three of these riboswitches positively regulate known or putative adhesins in response to c-di-GMP. Cdi-2-1 regulates CD630_32460, which encodes a protein predicted to localize to the bacterial surface. Cdi-2-3 regulates CD630_28310, a putative adhesin with a predicted collagen binding domain (33). Notably, the products of these genes have been shown to be sortase-dependent proteins cleaved by the ZmpI metalloprotease that is negatively regulated by c-di-GMP through Cdi-1-12 (33, 38). Cdi-2-4 controls expression of genes encoding TFP, which are involved in adherence to epithelial cells and intestinal colonization (32). Cdi-2-3 and Cdi-2-4 and their downstream genes are highly conserved in C. difficile genomes (Fig. S3). In contrast, Cdi-2-1 and the downstream CD630_32460 are present in approximately 2/3 of the genomes. Interestingly, though CD630_32670 is highly conserved in all of the available genomes, about 1/3 lack the upstream Cdi-2-2 riboswitch, suggesting alternative modes of regulation in some strains.

To validate the RNA-seq results, we performed qRT-PCR using RNA isolated from C. difficile with vector, pDccA, or pDccA^mut^ grown in the presence or absence of 1 µg/ml nisin. The pDccA^mut^ vector encodes a catalytically inactive form of DccA and allows us to attribute regulation specifically to increased c-di-GMP and not another potential function of the DccA protein (29). Four out of the seven genes adjacent to class I riboswitches showed decreased transcript levels in C. difficile pDccA (Table 2). Expression of all four of the genes 3′ of the class II c-di-GMP riboswitches was significantly increased in C. difficile with pDccA compared to the vector and pDccA^mut^ controls (Table 3). The average transcript levels for the other three genes was decreased in agreement with the RNA-seq data, but the differences did not meet statistical significance.

**TABLE 2:** Fold changes in transcripts for genes controlled by class I (GEMM) riboswitches as determined by qRT-PCR

Location and growth^*c*^	19900,^*a*^ Cdi-1-1	27970, Cdi-1-2	02450 (*flgB*), Cdi-1-3	19903, Cdi-1-8	23090, Cdi-1-9	33682, Cdi-1-11	28300 (*zmpI*), Cdi-1-12
Vector −	1	1	1	1	1	1	1
Vector +	1.09	1.46	1.21	0.81	0.85	0.61	0.85
pDccA^mut^ −	1.28	2.02	1.50	1.61	1.48	1.27	1.44
pDccA^mut^ +	1.27	1.82	1.57	2.04	2.12	1.25	0.90
pDccA −	**3.25**^*b*^	0.70	1.12	1.75	1.58	1.18	1.72
pDccA +	**149.50**	**0.24**	**0.18**	0.59	0.31	0.34	**0.15**

a Locus tag from *C. difficile* 630 genome sequence (GenBank accession no. AM180355.1) and upstream riboswitch.

b Bold numbers indicate values significantly different (*P* < 0.05) from *C. difficile* with vector grown without nisin, by 2-way ANOVA.

c +/− indicates cultures grown with or without 1 µg/ml nisin, respectively.

**TABLE 3:** Fold changes in transcripts for genes controlled by class II c-di-GMP riboswitches as determined by qRT-PCR

Location and growth^*c*^	32670,^*a*^ Cdi-2-1	32460, Cdi-2-2	28310, Cdi-2-3	35130 (*pilA1*), Cdi-2-4
Vector −	1	1	1	1
Vector +	0.87	0.75	0.99	0.82
pDccA^mut^ −	1.14	1.07	1.27	1.12
pDccA^mut^ +	1.16	0.76	0.88	0.60
pDccA −	1.25	1.88	2.43	2.22
pDccA +	**4.76**^*b*^	**17.35**	**40.86**	**18.44**

a Locus tag from *C. difficile* 630 genome sequence (GenBank accession no. AM180355.1) and upstream riboswitch.

b Bold numbers indicate values significantly different (*P* < 0.05) from *C. difficile* with vector grown without nisin, by 2-way ANOVA.

c +/− indicates cultures grown with or without 1 µg/ml nisin.

### Expression of genes downstream of predicted c-di-GMP riboswitches is altered in response to small changes in c-di-GMP.

The RNA-seq analysis was done following induction of *dccA* expression with 1 µg/ml nisin to ensure a robust increase in c-di-GMP and to maximize identification of regulated genes. However, previous studies found that this induction level increased intracellular c-di-GMP up to 2,000-fold, which is likely not a biologically meaningful change (29). We thus sought to determine the effect of more subtle changes in c-di-GMP on C. difficile gene expression. In addition, the presence of a large set of genes regulated (both positively and negatively) by c-di-GMP riboswitches provides the opportunity to examine the differences in responsiveness of this class of c-di-GMP receptor to changes in intracellular c-di-GMP. For example, differences between riboswitches of the same type may result in changes to ligand affinity, resulting in responses to different threshold concentrations of c-di-GMP (20, 39).

**FIG 2:**
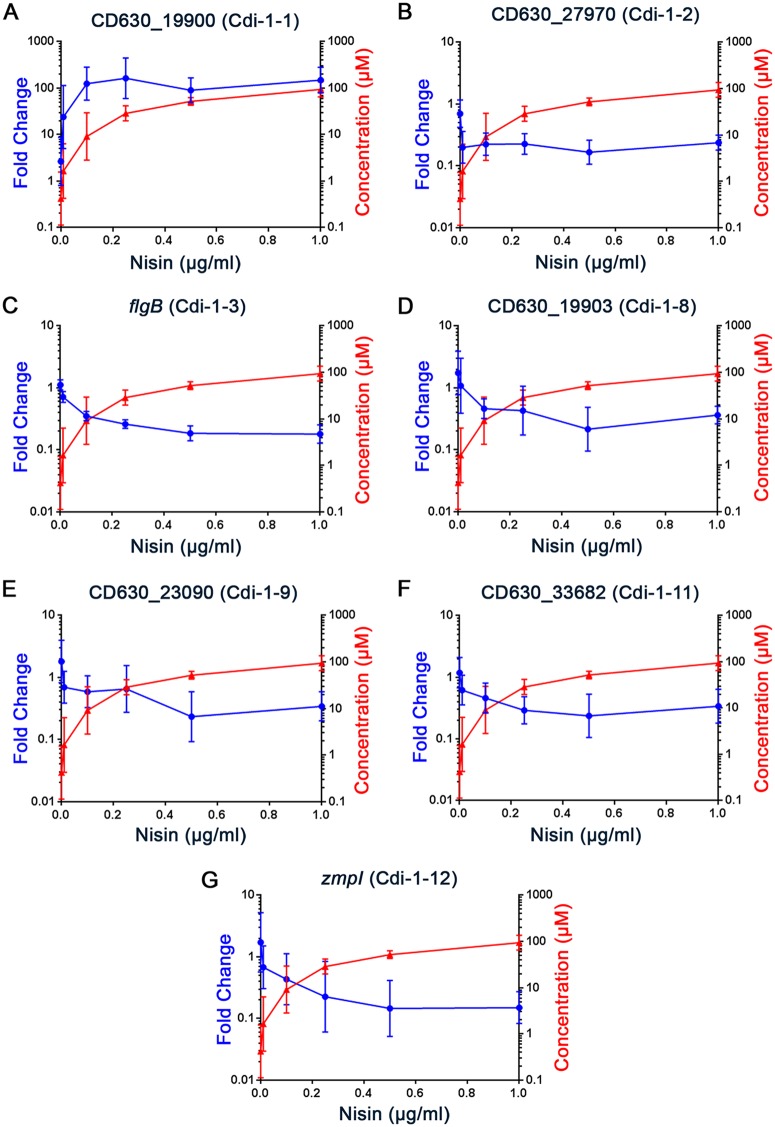
Regulation of genes downstream of class I (GEMM) riboswitches by c-di-GMP. C. difficile with vector or pDccA was grown with a range of nisin concentrations (µg/ml). Cultures were divided for quantification of intracellular c-di-GMP concentration by LC-MS (red) or measurement of transcript abundance for the downstream open reading frame by qRT-PCR (blue). Lines and error bars represent the geometric mean and geometric standard deviation. Not shown are those for which no downstream open reading frame or transcript was identified.

**FIG 3:**
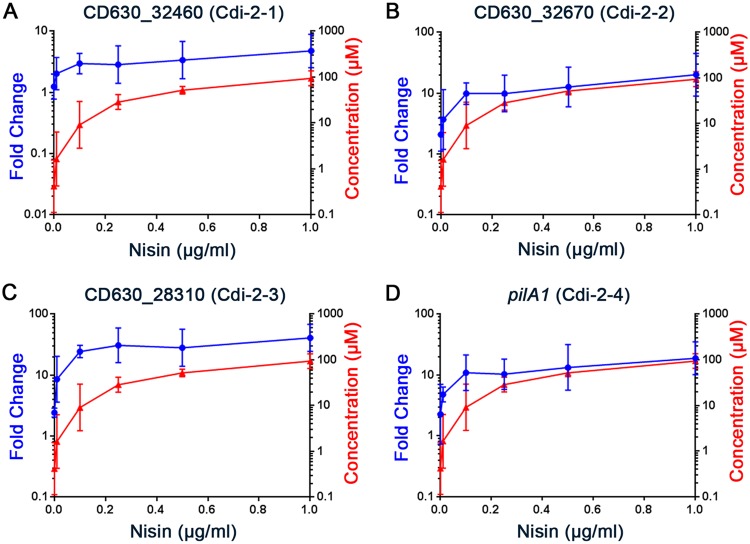
Genes 3′ of class II c-di-GMP riboswitches are positively regulated by c-di-GMP. C. difficile with vector or pDccA was grown with a range of nisin concentrations (µg/ml). Cultures were split for quantification of intracellular c-di-GMP concentration by LC-MS (red) or measurement of transcript abundance for the downstream open reading frame by qRT-PCR (blue). Lines and error bars represent the geometric mean and geometric standard deviation.

To evaluate the responsiveness of the riboswitches to c-di-GMP, we measured the transcript abundance of the gene immediately 3′ of each riboswitch in C. difficile with a range of intracellular c-di-GMP concentrations. To achieve a range of intracellular c-di-GMP concentrations, C. difficile pDccA was grown with nisin inducer in concentrations ranging from 0 to 1 μg/ml. This range of nisin concentrations resulted in a dose-dependent decrease in swimming motility of the C. difficile pDccA strain, with 0.1 µg/ml resulting in a downward trend in motility and 0.25 µg/ml significantly inhibiting motility (Fig. S4). As controls, C. difficile with vector or pDccA^mut^ was grown with 0 or 1 µg/ml nisin. The cultures were divided, with one portion used for nucleotide extraction and measurement of intracellular c-di-GMP concentration by UPLC-MS, and the other portion used for RNA extraction and evaluation of transcript abundance by qRT-PCR. The control strains contained low intracellular c-di-GMP concentrations (131 nM ± 268 nM) regardless of nisin addition, consistent with previously reported data for C. difficile (29). Induction of *dccA* with increasing levels of nisin led to a dose-dependent increase in c-di-GMP, yielding a near-linear 156-fold range from 630 nM with no nisin to 99 µM at the highest nisin concentration (Fig. 2 and 3 and Fig. S5). The corresponding effects of c-di-GMP on transcript abundance for the downstream gene were expressed as the fold change compared to vector control grown without nisin. We note that the nonnormalized abundances are affected by the strength of the respective promoters and vary greatly between the loci (Fig. S6).

10.1128/mSphere.00423-18.4 FIG S4 Dose-dependent decrease in C. difficile motility in response to c-di-GMP. C. difficile 630Δerm with pDccA or vector was assayed for motility in 0.5× BHIS-0.3% agar medium supplemented with 10 µg/ml Tm, and the indicated concentrations of nisin to induce *dccA* expression and c-di-GMP biosynthesis. Diameters of motility were measured after 48 hours of growth at 37°C. Shown are the means and standard deviations for 4 independent replicates. **, *P* < 0.01; ***, *P* < 0.001, by unpaired *t* test. Download FIG S4, PDF file, 0.2 MB. Copyright © 2018 McKee et al. 2018 McKee et al. This content is distributed under the terms of the Creative Commons Attribution 4.0 International license.

10.1128/mSphere.00423-18.5 FIG S5 Change in intracellular c-di-GMP in response to induction of *dccA* expression with nisin. C. difficile pDccA was grown with 0, 0.01, 0.1, 0.25, 0.5, or 1.0 µg/ml nisin, and then nucleotides were extracted for quantification of c-di-GMP by UPLC-MS. Data are expressed as concentration of c-di-GMP in cell volume extracted as described previously (5, 7). Shown are the means and standard errors for three independent samples. Download FIG S5, PDF file, 0.2 MB. Copyright © 2018 McKee et al. 2018 McKee et al. This content is distributed under the terms of the Creative Commons Attribution 4.0 International license.

10.1128/mSphere.00423-18.6 FIG S6 The effect of artificially increased c-di-GMP on riboswitch gene expression. Shown are the means and standard deviations of the reads normalized per kilobase per million reads (RPKM) for genes downstream of c-di-GMP riboswitches. All meet *P* < 0.05 after Bonferroni’s correction. Download FIG S6, PDF file, 0.2 MB. Copyright © 2018 McKee et al. 2018 McKee et al. This content is distributed under the terms of the Creative Commons Attribution 4.0 International license.

For most of the genes, transcript abundance was significantly different between C. difficile pDccA grown with 0.1 µg/ml nisin and C. difficile with vector grown without nisin (Fig. 2 and 3). These conditions correspond to a 19-fold difference in intracellular c-di-GMP, as well as a detectable decrease in swimming motility (see Fig. S4 and S5). Increasing nisin concentrations above 0.1 µg/ml generally resulted in modest additional effects on the expression of these genes, though there were differences in the range of responses to c-di-GMP. For example, the abundances of the CD630_27970, CD630_19903, CD630_23090, and CD630_33682 transcripts decreased between 0, 0.01, and 0.1 µg/ml nisin (632 nM, 2.54 µM, and 13.67 µM c-di-GMP, respectively) and then remained constant up to 1 µg/ml nisin (99 µM c-di-GMP) (Fig. 2). In contrast, the transcript abundances of *flgB* and *zmpI* continued to decrease with increasing c-di-GMP. One possible explanation for these differences is that the baseline gene expression varies, affecting the number of target RNA molecules available for saturation of binding by c-di-GMP. However, despite dissimilar expression kinetics in response to c-di-GMP, CD630_27970, *flgB*, and *zmpI* showed comparable baseline transcript levels (Fig. S6). These differences reflect potential variability in the responsiveness of class I riboswitches to c-di-GMP.

Regulation through class II c-di-GMP riboswitches similarly shows the greatest response between 632 nM (baseline), 2.54 µM, and 13.67 µM c-di-GMP (Fig. 3). CD640_32670, CD630_28310, and *pilA1* increased 4.4-, 10.1-, and 3.9-fold in this range and reached 11.7-, 18.6-, and 10.8- fold increases in C. difficile with 99 µM c-di-GMP, respectively. CD630_32460 transcript abundance increased more gradually, reaching a maximum 4.9-fold increase in C. difficile with 99 µM c-di-GMP. Taken together, these results indicate that gene regulation via c-di-GMP riboswitches occurs in response to small and likely physiologically relevant changes in c-di-GMP *in vivo*.

### Regulation of CD630_19900 expression by c-di-GMP occurs at multiple levels.

Based on RNA-seq and qRT-PCR analyses, class I c-di-GMP riboswitches in C. difficile appeared to function as “off” switches with the exception of Cdi-1-1, for which expression of the downstream CD630_19900 gene increased in response to c-di-GMP. However, recent work on the Vc2 c-di-GMP riboswitch in Vibrio cholerae demonstrated that c-di-GMP can impact expression of a gene at multiple levels (40). In the case of Vc2, positive regulation by c-di-GMP via the promoter could override inhibition of expression through Vc2, leading to an observed increase in gene expression in response to c-di-GMP. To address the possibility that c-di-GMP also regulates transcription initiation of CD630_19900, and possibly other genes, we generated transcriptional fusions of the alkaline phosphatase (AP) reporter gene *phoZ* to the regions encompassing each of the promoters but excluding the riboswitches. The fragments consisted of ∼200 bp encompassing the promoter (location estimated based on RNA-seq read mapping) and at most 10 bases of the 5′ end of the riboswitch sequences such that no functional riboswitch is present, allowing us to specifically determine the extent of c-di-GMP regulation via the promoters. These fusions were integrated into the C. difficile 630Δerm chromosome, and then pDccA or vector was introduced. The strains were grown with a range of nisin concentrations to induce *dccA* expression and c-di-GMP production, and then AP activity was assayed as previously described (41).

**FIG 4:**
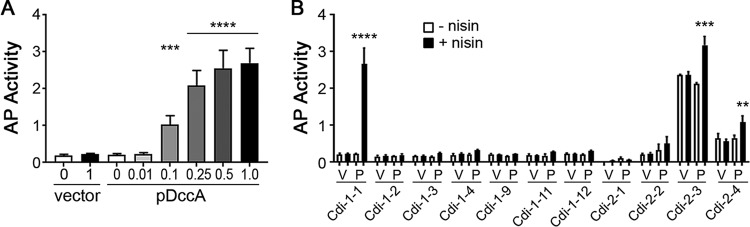
Alkaline phosphatase reporter assays of riboswitch-adjacent gene promoters. C. difficile 630Δerm bearing promoter-*phoZ* reporter fusions and either vector or pDccA was grown to mid-exponential phase with the indicated concentration of nisin (µg/ml). (A) Reporter activity for C. difficile with the Cdi-1-1 (CD630_19900) promoter fusion, grown with a range of nisin concentrations. (B) Reporter activity for C. difficile with fusions to the promoter regions upstream of the indicated riboswitches (but lacking the riboswitches themselves), grown with or without 1 µg/ml nisin (black bars and white bars, respectively). The means and standard deviations of 3 biological replicates are shown. **, *P* < 0.01; ***, *P* < 0.001; and ****, *P* < 0.0001, using one-way ANOVA and Dunnett’s posttest, compared to the induced vector control for the respective reporter fusion.

For the CD630_19900 promoter fusion, AP activity increased in response to nisin in a dose-dependent manner, up to a 13-fold increase in activity between the lowest and highest c-di-GMP levels tested (Fig. 4A). These results indicate that transcription initiation is positively regulated by c-di-GMP. c-di-GMP may still inhibit transcription read-through via the riboswitch, but the increased expression from the promoter is dominant under these conditions. Fusions to promoter regions for the remaining loci showed no change or up to a 2-fold change in expression at the highest concentration of c-di-GMP (Fig. 4B), suggesting that c-di-GMP does not substantially affect their transcription initiation.

### c-di-GMP broadly regulates the composition of the C. difficile cell surface to influence surface interactions.

**FIG 5:**
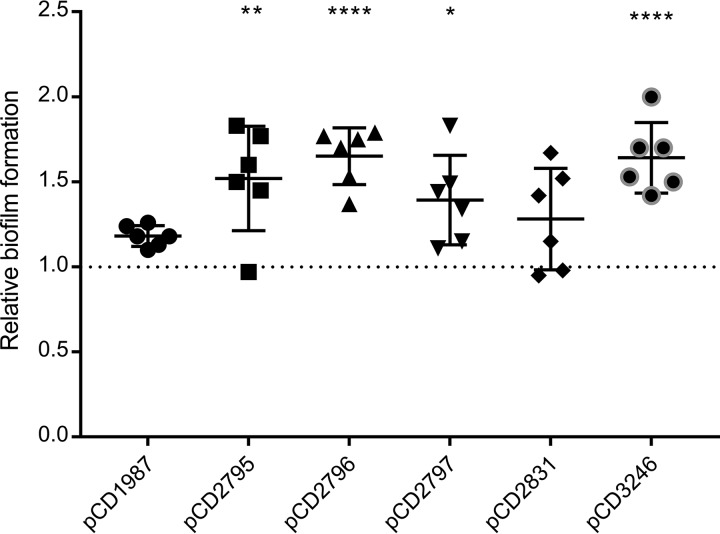
Expression of genes encoding cell envelope proteins promotes biofilm formation. Genes encoding putative cell envelope proteins were expressed under the control of an ATc-inducible promoter in C. difficile 630Δerm. The strains were grown in 24-well plates in buffered BHIS containing 1% glucose and 20 ng/ml ATc to induce gene expression during biofilm development. Biofilm formation was assayed by crystal violet after 24-h incubation. Values are normalized to the induced vector control. Symbols indicate values from independent biological replicates, and bars indicate the means and standard deviations for six independent replicates. *, *P* < 0.05; **, *P* < 0.01; and ****, *P* < 0.0001, by 1-way ANOVA and Dunnett’s posttest, compared to the vector control.

The RNA-seq analysis supported prior studies showing that c-di-GMP negatively regulates the production of flagella by inhibiting flagellar gene expression, and positively regulates type IV pilus biosynthesis by promoting TFP gene expression. In addition, c-di-GMP positively regulates CD630_28310 and CD630_32460, which encode sortase-dependent surface proteins, and inhibits expression of the ZmpI zinc-dependent metalloprotease that cleaves them (21, 33). Transcriptional analysis identified additional c-di-GMP-regulated candidate surface proteins involved in C. difficile adherence: CD630_19870, CD630_27950, CD630_27960, and CD630_27970. While the last three are adjacent on the chromosome, they are divergently transcribed and do not compose an operon. The fold changes in transcript levels for these six genes under elevated c-di-GMP conditions, based on the RNA-seq analysis, are listed in Table 4. We speculated that one or more of these proteins plays a role in biofilm formation, a process that is promoted by c-di-GMP in C. difficile (31, 35). To test this, genes encoding the individual cell envelope proteins were expressed ectopically in C. difficile from an ATc-inducible promoter (P*_tet_*) during growth in static culture for 24 h to allow biofilm development (31). Four of the six genes (CD630_27950, CD630_27960, CD630_27970, and CD630_32460) significantly increased biofilm formation compared to the vector control (Fig. 5). The remaining two genes (CD630_19870 and CD630_28310) also increased biofilm formation somewhat, but the results did not achieve statistical significance.

**TABLE 4:** Putative cell envelope proteins whose expression is regulated by c-di-GMP

Locus	Fold change^*a*^ (pDccA/vector)	Predicted function	Riboswitch upstream?
CD630_19870	**4.32**	Cell wall protein 28	No
CD630_27950	**2.90**	Cell wall protein 11	No
CD630_27960	*−4.06*	Cell wall protein 10	No
CD630_27970	*−7.15*	Calcium-binding adhesion protein	Yes
CD630_28310	**42.51**	Putative adhesin	Yes
CD630_32460	**4.29**	Surface protein	Yes

a Boldface and italic indicate significantly increased and decreased abundance relative to the vector control, respectively, as determined using RNA-seq.

## DISCUSSION

In this work, we set out to define the transcriptional regulon of c-di-GMP in the human pathogen C. difficile. We found that c-di-GMP regulates a total of 166 genes under the growth conditions tested. While many of these genes were previously identified members of the c-di-GMP regulon in C. difficile (29, 30, 33), numerous additional c-di-GMP-regulated cellular processes were identified. Known and putative colonization factors were prominent in the regulon, consistent with a role for c-di-GMP in adherent behaviors of C. difficile (29–33).

Genes directly regulated via c-di-GMP riboswitches were highly represented, including 29 flagellar genes contained in the 23 kb *flgB* operon directly regulated by Cdi-1-3 and the TFP genes directly regulated by Cdi-2-4. For many of the remaining genes, the mechanism of regulation by c-di-GMP is unclear. The only predicted transcriptional regulators identified were SigD and two putative response regulators containing DNA binding domains (CD630_32650 and CD630_32670). These three regulators are encoded in operons directly controlled by c-di-GMP riboswitches and could relay changes in intracellular c-di-GMP to modulate expression of other loci. For example, SigD coordinates the indirect activation of flagellar genes carried outside the *flgB* operon, as well as a number of other genes involved in metabolism, membrane transport, and toxin biosynthesis (8, 29, 42). The partial overlap between the c-di-GMP regulon and the previously reported SigD regulon indicates the subset of genes controlled by the SigD sigma factor (42). The response regulators encoded by CD630_32650 and CD630_32670 likely mediate c-di-GMP regulation for additional genes. In addition, although no protein receptors of c-di-GMP have been identified in C. difficile to date, it is possible that c-di-GMP posttranslationally regulates the function of proteins that in turn modulate gene expression, as described in other species (18, 19, 43).

Of the 16 predicted c-di-GMP riboswitches in C. difficile, 11 are carried near genes on the same coding strand, positioning them to regulate the expression of these downstream genes. Consistent with this, expression of these 11 genes was altered by increasing the c-di-GMP concentration *in vivo* in a dose-dependent manner, with a 19-fold increase in c-di-GMP resulting in a significant change in gene expression. The normal dynamic range of c-di-GMP in C. difficile in response to extracellular stimuli is unknown, so it is unclear whether the 19-fold increase reflects a biologically meaningful change. Motility assays showed that a comparable level of *dccA* induction results in detectable changes in C. difficile swimming *in vitro*. These results indicate that even small increases in c-di-GMP are capable of altering gene expression, with consequent effects on C. difficile physiology and behavior. One limitation of our study is that it was not possible to determine quantitatively the effects of reducing c-di-GMP on gene expression, as basal c-di-GMP levels present in the control strain were already near the limit of detection. In addition, the measurements described averaged c-di-GMP concentrations and transcript levels across the population. Prior work in C. difficile and other species suggests heterogeneity in c-di-GMP levels, and concomitantly in gene expression, among individual bacteria (44–47). Thus, measurements of c-di-GMP and mRNA in bulk populations may under- or overestimate the responses of single cells.

Initial transcriptional analyses indicated that class II riboswitches are uniformly “on” switches, while all but one class I riboswitch are “off” switches in response to c-di-GMP in C. difficile. However, there are multiple examples of ligands also affecting the activity of the promoter upstream of their cognate riboswitch (39, 40, 48, 49). For example, *in vivo* studies of class I c-di-GMP riboswitches in Vibrio cholerae, Vc1 and Vc2, showed that c-di-GMP regulates the expression the downstream genes *gbpA* and *tfoY* at two levels: by controlling transcription initiation via the upstream promoters and via the riboswitches (39, 40). Moreover, the two control mechanisms acted in opposing directions (39, 49). Using reporter fusions to the regions upstream of the functional c-di-GMP riboswitches in C. difficile, we found that c-di-GMP controls expression of CD630_19900 via the promoter. These results may explain the observation that CD630_19900 is overall positively regulated by c-di-GMP; despite being preceded by a class I riboswitch, which presumably functions as an “off” switch, regulation via the promoter supersedes riboswitch control under the conditions tested. Thus, while *in vitro* transcription can be used to measure interactions between c-di-GMP and mRNA, these interactions need to be placed in the appropriate biological context before making conclusions about c-di-GMP regulation *in vivo*.

Of six genes encoding cell wall-localized proteins, four significantly increased *in vitro* biofilm formation when overexpressed. Despite negative regulation by c-di-GMP, CD630_27960 and CD960_27970 increased biofilm formation when overexpressed. We speculate that overexpression caused unexpected changes to the composition of the cell surface or overall bacterial physiology, resulting in more adherent bacteria independent of the individual gene expressed. Ongoing studies examining mutants in these genes, individually and in combination, will determine the roles of these loci in the surface behaviors of C. difficile. Several studies have described C. difficile biofilm development *in vitro* (50–54), and a few microbial factors that contribute to this process have been reported, including cell surface proteins, their regulators, and more (31, 35, 55–62). Some of the factors involved in biofilm formation include surface proteins regulated by c-di-GMP, including TFP, flagellin, CD630_28310, and CD630_32460 (31, 55, 56, 63). The contribution of biofilm formation to C. difficile disease remains largely speculative. *In vitro* biofilms are reservoirs for spores, accumulate toxins, and show increased antibiotic tolerance, suggesting that biofilms may aid in persistent colonization and disease recurrence (51, 56, 64). Recent work using animal models supports that C. difficile is present in microbial communities with biofilm-like characteristics (65, 66). Future studies examining factors required for biofilm development, including those regulated by c-di-GMP, could be used to determine the importance of biofilms to C. difficile disease.

In summary, this work expands the known c-di-GMP signaling network, demonstrates the functionality of c-di-GMP riboswitches in this organism, and highlights the role of the riboswitches in controlling known and putative virulence factors in C. difficile.

## MATERIALS AND METHODS

### Bacterial growth conditions.

C. difficile cultures were grown at 37°C in an atmosphere of 5% CO_2_, 5% H_2_, and 90% N_2_ using a Coy anaerobic chamber. Overnight cultures of C. difficile were grown in 2 to 5 ml of TY medium (30 g/liter Bacto tryptone, 20 g/liter yeast extract, 1 g/liter thioglycolate) with antibiotics as necessary for maintenance of plasmids. For experiments, overnights cultures were diluted in BHIS medium (37 g/liter brain heart infusion, 5 g/liter yeast extract). Antibiotics were used at the following concentrations: thiamphenicol (Tm), 10 μg/ml; chloramphenicol (Cm), 10 μg/ml; ampicillin (Amp), 100 μg/ml; and kanamycin (Kn), 100 μg/ml. Nisin was added at 0.01 to 2.0 µg/ml as indicated to induce expression from the *cpr* promoter. Strains and plasmids used in this study are described in Table S1 in the supplemental material.

10.1128/mSphere.00423-18.7 TABLE S1 Strains and plasmids used in this study. Download Table S1, PDF file, 0.7 MB. Copyright © 2018 McKee et al. 2018 McKee et al. This content is distributed under the terms of the Creative Commons Attribution 4.0 International license.

### RNA sequencing.

Single colonies of 630Δerm bearing vector or pDccA were inoculated in TY-Tm medium and grown for ∼16 h, with 4 independent replicates per strain. Cultures were diluted 1:100 in 5 ml of BHIS-Tm and grown to an optical density at 600 nm (OD_600_) of 0.2. Nisin was then added to each culture at a final concentration of 1 μg/ml. Cultures were then grown to an OD_600_ of 1.0. Cells were collected by centrifugation at 3,000 × *g* for 10 min. Supernatants were removed, and RNA was extracted using TriSure (Bioline) and chloroform as described previously (29, 55). RNA was precipitated with 100% isopropanol and centrifugation at 13,000 × *g* for 10 min at 4°C. Supernatants were removed, and RNA pellets were washed with cold 70% ethanol and subjected to centrifugation at 13,000 × *g* for 10 min at 4°C. Air-dried RNA pellets were suspended in 50 μl of nuclease-free water. RNA was treated with Turbo DNA-free (Ambion) to remove DNA, and RNA integrity was checked using a Bioanalyzer assay. All RNA samples had RNA integrity numbers greater than 8. rRNA was depleted using a Ribo Zero (bacteria) rRNA removal kit (Illumina). Libraries were prepared using the TruSeq Ribo Zero Gold kit (Illumina). Samples were pooled for single-end sequencing on a Hi-Seq 2500 sequencer (Illumina). A combined 46.9 million (46.9M) 50-nt reads were obtained for vector control samples, and 63.9M 50-nt reads for pDccA samples. Base calling and demultiplexing of the data were done using Illumina bcl2fastq v.2.17.0.

RNA sequencing analysis was performed using CLC Genomic Workbench version 9.0 (Qiagen). Transcripts were mapped to the C. difficile 630 genome (AM180355.1), on which annotations for the c-di-GMP riboswitches were manually added based on the predictions by Sudarsan et al. and Lee et al. (20, 21). Reads were mapped to the reference genome with the software’s default scoring penalties for mismatch, deletion, and insertion differences from the reference genome. Transcript reads for each gene were normalized to the total number of reads and gene length (expressed as reads per kilobase of transcript, per million mapped reads [RPKM]) before calculating the fold change. Fold decreases are expressed as negative numbers. Genes were considered to be regulated by c-di-GMP if the fold change between the means of the pDccA- and vector-bearing strains was greater than 2 and *P* < 0.05 following Bonferroni correction for multiple comparisons.

### qRT-PCR.

Cultures of C. difficile 630Δerm bearing vector, pDccA, or pDccA^mut^ were grown as above but in a 25-ml volume of BHIS to allow splitting of the cultures for c-di-GMP quantification (see below) and quantitative reverse transcription-PCR (qRT-PCR). Cultures were grown with a range of nisin concentrations to achieve a range of *dccA* expression and intracellular c-di-GMP levels (29). For the vector and pDccA^mut^ control strains, two nisin concentrations, 0 µg/ml and 1 µg/ml, were used. For the pDccA strain, 7 concentrations were used: 0, 0.01, 0.1, 0.25, 0.5, 1.0, and 2.0 µg/ml. The cultures were grown to exponential phase (OD_600_ of 1.0), and 3-ml samples were collected for qRT-PCR. Following RNA purification as above, cDNA was synthesized using the High Capacity cDNA Reverse Transcription kit (Applied Biosystems). A total of 10 ng of cDNA template was used in each qRT-PCR with SensiMix SYBR Green (Bioline). Primers, designated as gene-qF and qR for the forward and reverse primers, respectively, were used at a final concentration of 300 nM. All PCR primers were designed to yield comparably sized products from the first 500 bp (or less) of the gene, and all sets were determined to have >95% amplification efficiency. The data were analyzed using the 2^−ΔΔ^*^CT^* method, with *rpoC* as a reference gene and normalization to the stated reference condition or strain (29, 55, 67).

### Quantification of intracellular c-di-GMP.

The remaining 22 ml of the above cultures was collected as matched samples for c-di-GMP quantification. Serial dilutions were plated in BHIS agar for enumeration of CFU. Cells were then collected by centrifugation at 2,500 × *g* for 10 min. Supernatants were removed, and cell pellets were suspended in 1 ml PBS and transferred to a 1.5 -ml microcentrifuge tube. Samples were centrifuged at 10,000 × *g*, and supernatants were removed. Pellets were suspended in 200 μl of extraction buffer (40% acetonitrile, 40% methanol, 0.1 N formic acid) and placed at −20°C for 30 min. Samples were centrifuged at 12,000 × *g* for 5 min at 4°C, and 200-μl aliquots of the supernatant were transferred to clean tubes and immediately neutralized by adding 8 μl of 15% (wt/vol) NH_4_HCO_3_. The c-di-GMP concentration in these samples was determined by UPLC/MS as described previously (55). Intracellular c-di-GMP was calculated by normalizing measured c-di-GMP concentrations to the total cytoplasmic volume extracted, estimated using enumerated CFU in each sample, as described previously (29, 55).

### Construction of plasmids for expression of genes encoding cell wall proteins.

Primers used for strain construction are listed in Table S2 in the supplemental material. The *tet* promoter from pRPF185 (68) was amplified by PCR using primers ATc_F + ATc_R. The PCR product was digested with the restriction enzymes EcoRI and SacI, ligated into similarly digested pMC123, and transformed into Escherichia coli DH5α. This plasmid (pRT1648) served as the vector control. The cell wall protein genes were amplified from 630Δerm genomic DNA using primers named according to LOCUSTAG_F and LOCUSTAG_R for the forward and reverse primers, respectively. The PCR products were ligated into pRT1648 using the SacI and BamHI restriction sites. These plasmids were conjugated into C. difficile 630Δerm as described previously (29).

10.1128/mSphere.00423-18.8 TABLE S2 Oligonucleotides used in this study. Download Table S2, PDF file, 0.5 MB. Copyright © 2018 McKee et al. 2018 McKee et al. This content is distributed under the terms of the Creative Commons Attribution 4.0 International license.

10.1128/mSphere.00423-18.9 TABLE S3 Genes regulated by c-di-GMP in C. difficile. Download Table S3, PDF file, 0.6 MB. Copyright © 2018 McKee et al. 2018 McKee et al. This content is distributed under the terms of the Creative Commons Attribution 4.0 International license.

### Biofilm assay.

Biofilm formation was assayed as described previously (31, 32, 55). Briefly, biofilms were grown in 1 ml modified BHIS (BHIS + 1% glucose + 50 mM sodium phosphate, pH 7.5) containing 10 µg/ml thiamphenicol and 20 ng/ml ATc. Bacteria were grown statically for 24 h at 37°C anaerobically. After 24 h, biofilm formation was measured using crystal violet staining.

### Motility assay.

Swimming motility was assayed as described previously (29, 44). Briefly, 2 µl of overnight cultures were inoculated into 0.5× BHIS-0.3% agar medium supplemented with Tm10 to maintain plasmids and incubated at 37°C. Diameters of motility were measured after 24 h.

### Construction of reporter fusions.

Promoter regions upstream of each riboswitch were amplified from 630Δerm genomic DNA using primers named cdiX-XpromF and cdiX-XpromR (Table S2). For example, for Cdi-1-1, the promoter was amplified using Cdi1-1promF and Cdi1-1promR. Amplified promoter regions were cloned into SalI- and SphI-digested pRT1346 (pSMB47::*phoZ*) (44) and then transformed into E. coli DH5α cells. Correct clones were identified by PCR and sequencing. Purified plasmids were transformed into Bacillus subtilis BS49, which contains Tn*916* (69, 70). Single colonies of the BS49 strains containing the reporter fusions were used in conjugations with C. difficile 630Δerm as described previously (44, 71). This method results in semirandom integration of the reporter fusion on the C. difficile chromosome. Plasmids pMC-Pcpr (vector) and pDccA were introduced into these C. difficile strains via conjugation with E. coli HB101(pRK24) donors (29).

### Alkaline phosphatase assay.

Single colonies of reporter fusion strains bearing vector or pDccA were inoculated into 2 ml TY-Tm and grown for ∼16 h. Cultures were diluted 1:100 in 3 ml of BHIS-Tm with various concentrations of nisin (0, 0.01, 0.1, 0.25, 0.5, 1.0, and 2.0 μg/ml). At mid-exponential phase (OD_600_ of ∼1.0), 1 ml of each culture was collected, and cells were collected by centrifugation for 1 min at 12,000 × *g*. Supernatants were discarded, and bacteria were stored at −20°C. Alkaline phosphatase activity was measured as described by Edwards et al. (41).

### Accession number(s).

The raw and processed data files are accessible through NCBI Geo (accession number GSE120198).
