# Supplementary figures and images for: Cyclic Diguanylate Regulates Virulence Factor Genes via Multiple Riboswitches in Clostridium difficile
Source: mSphere. 2018 Oct 24;3(5):e00423-18. doi: 10.1128/mSphere.00423-18 (PMC6200980; doi:10.1128/mSphere.00423-18)

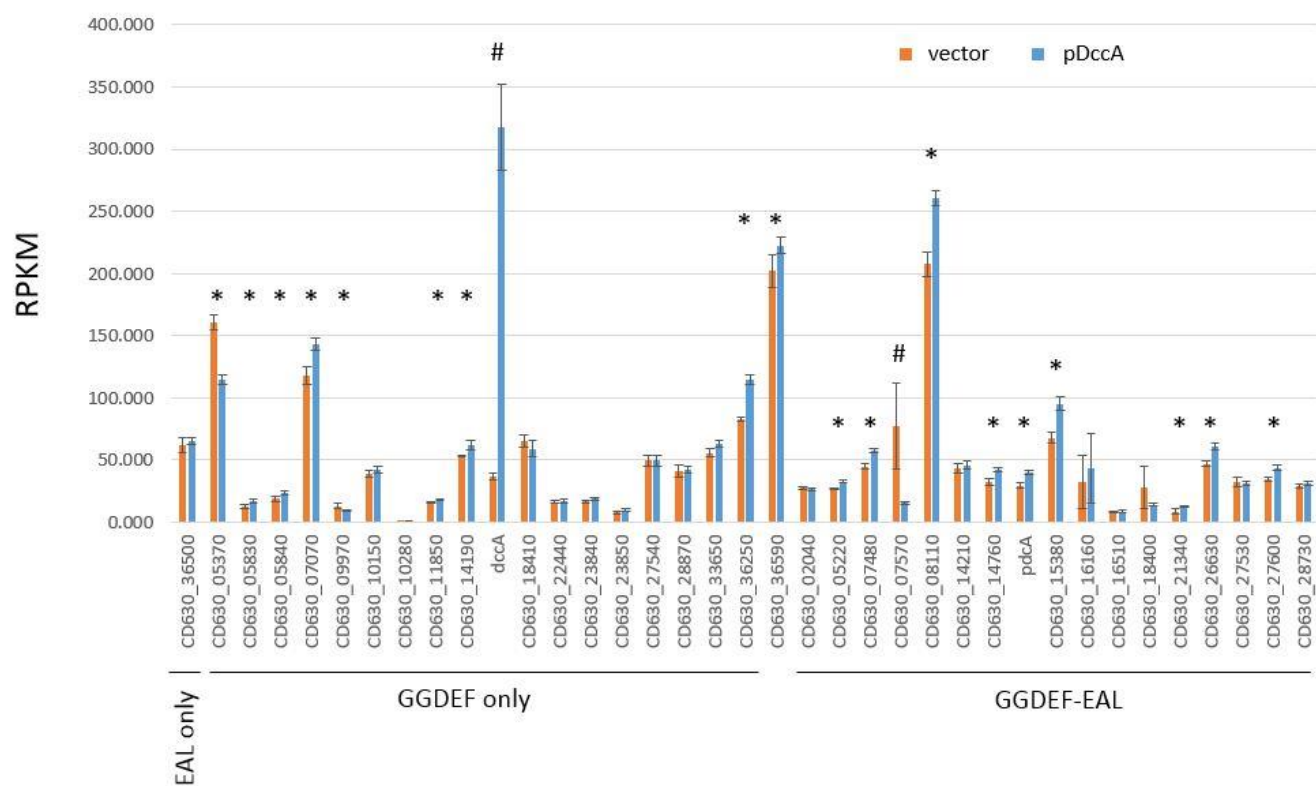

**Figure S1**

Supplement: FIG S1 [file sph005182673sf1.pdf]

Figure S2

Cdi-1-4

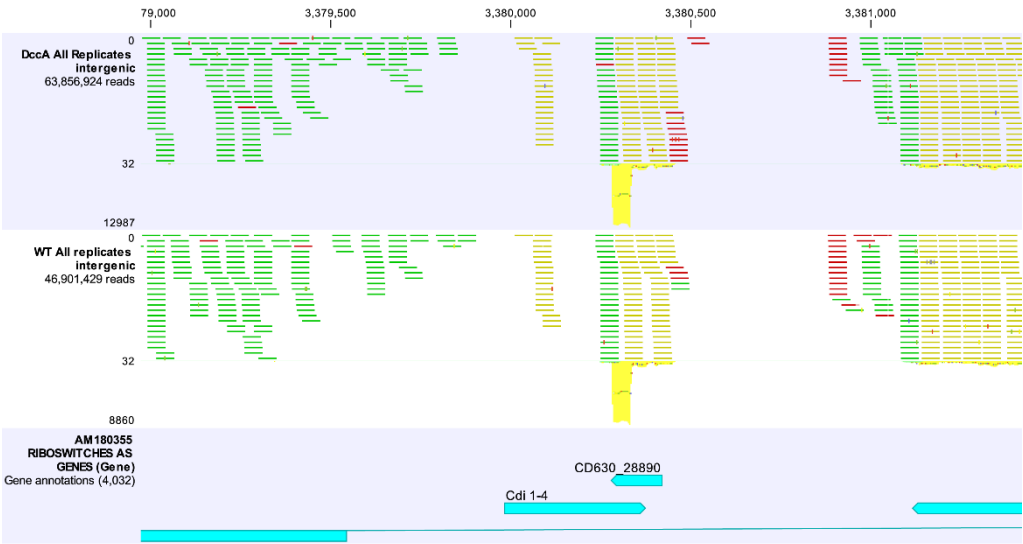

Cdi-1-5

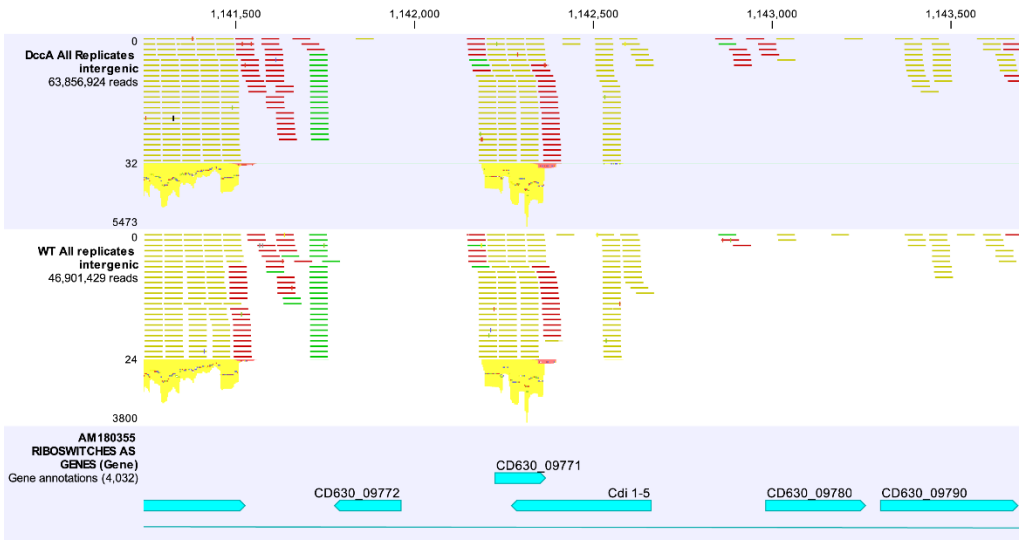

Cdi-1-6

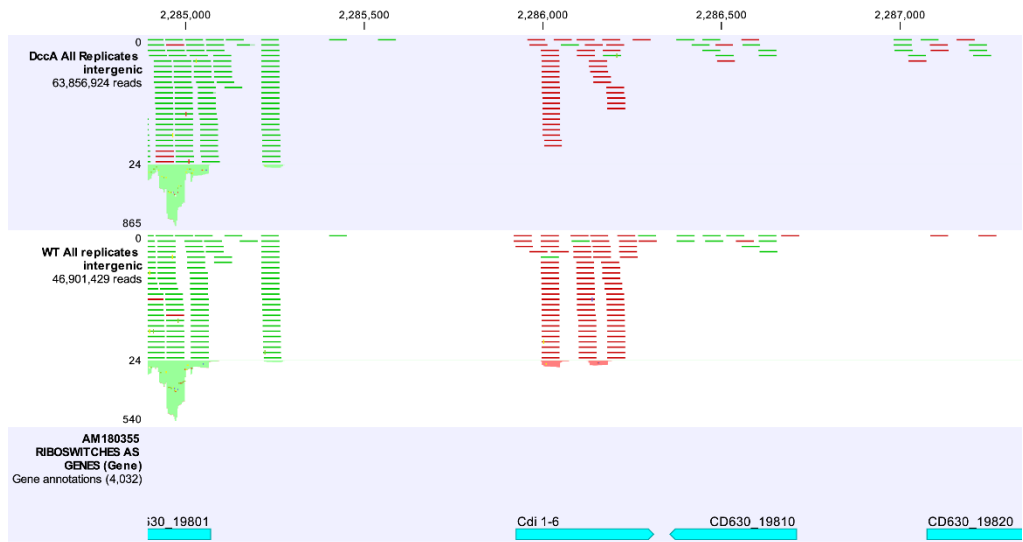

Cdi-1-7

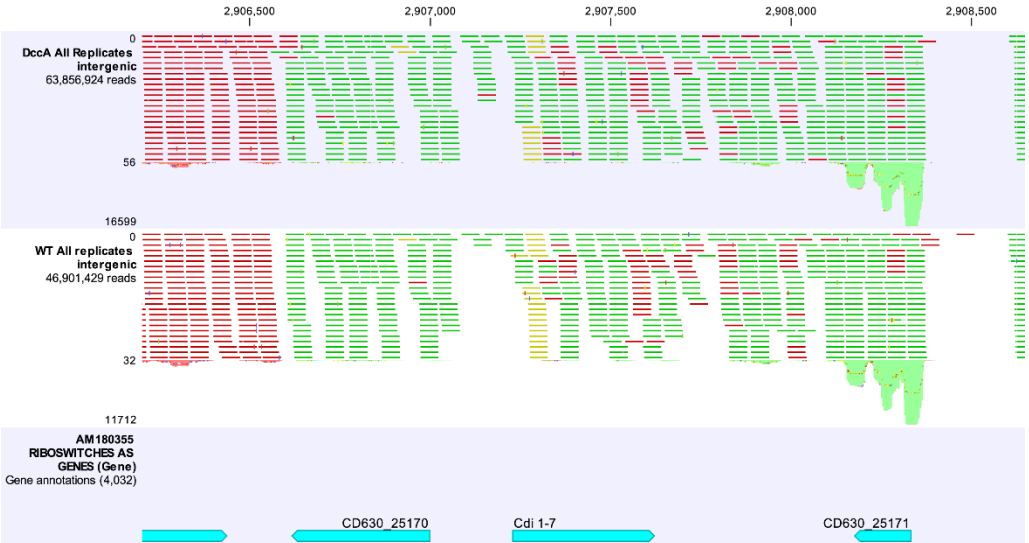

Cdi-1-10

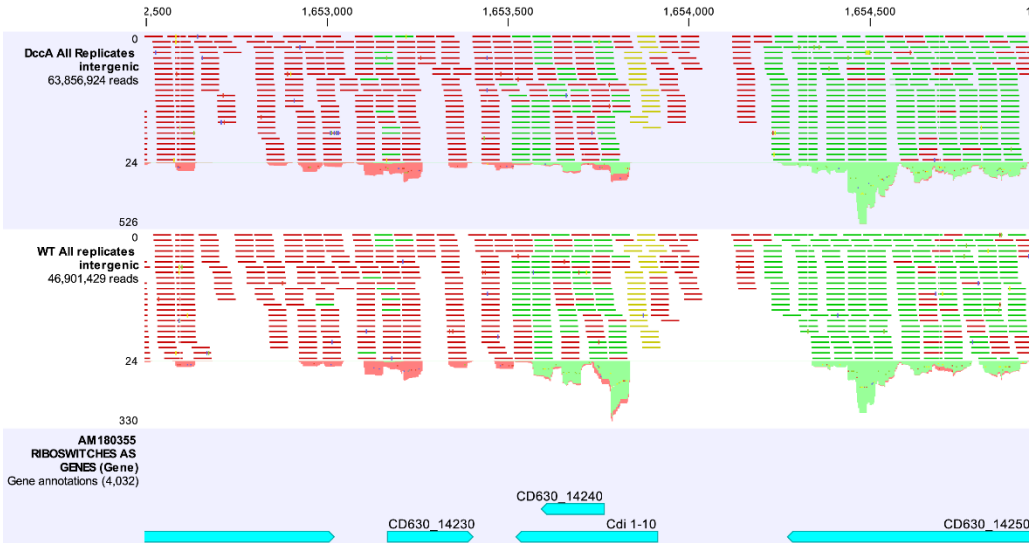

Supplement: FIG S2 [file sph005182673sf2.pdf]

Figure S4

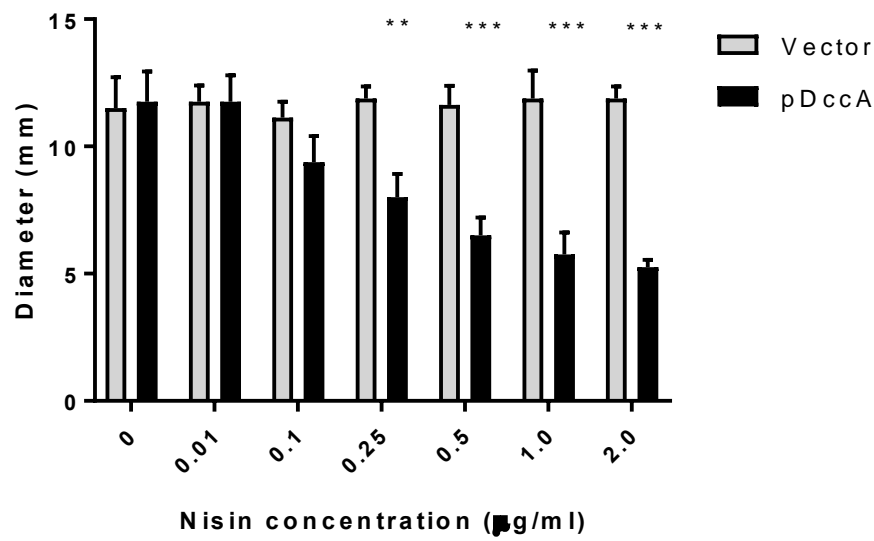

Supplement: FIG S4 [file sph005182673sf4.pdf]

Figure S5

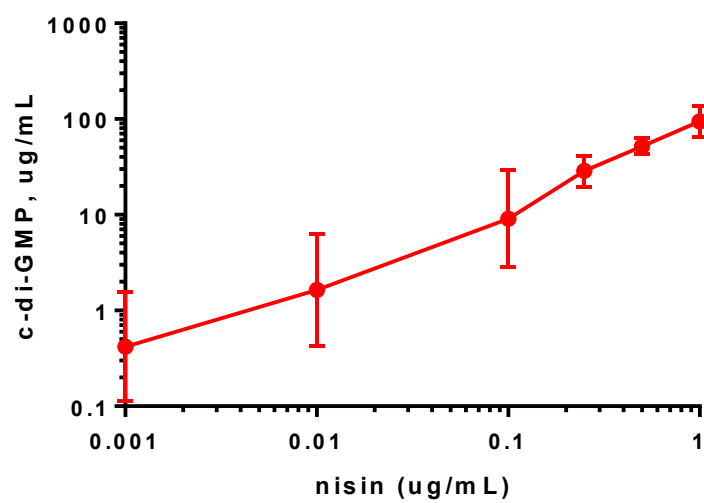

Supplement: FIG S5 [file sph005182673sf5.pdf]

Figure S6

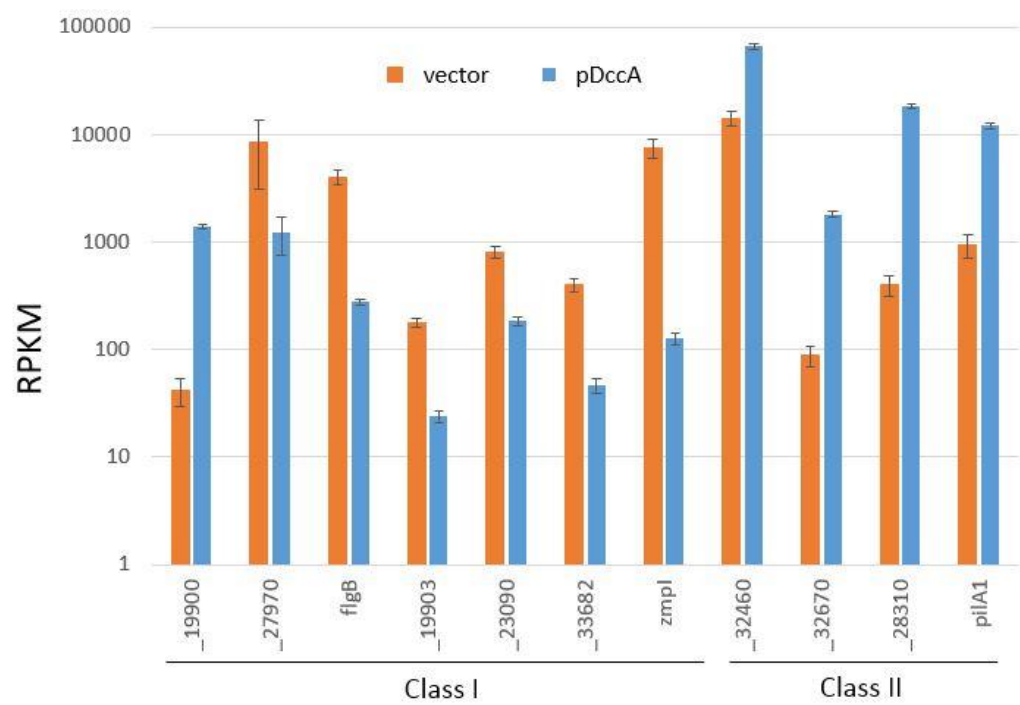

Supplement: FIG S6 [file sph005182673sf6.pdf]
