# Supplementary material for: Cyclic Diguanylate Regulates Virulence Factor Genes via Multiple Riboswitches in Clostridium difficile
Source: mSphere. 2018 Oct 24;3(5):e00423-18. doi: 10.1128/mSphere.00423-18 (PMC6200980; doi:10.1128/mSphere.00423-18)
Supplement: FIG S3 [file sph005182673sf3.pdf]

Figure S3

Accession	Strain/Description	Riboswitch																	1-3	2-1	2-2
		1-1	1-2	1-3	1-4	1-5	1-6	1-7	1-8	1-9	1-10	1-11	1-12	2-1	2-2	2-3	2-4		0245	3246	3267
CP011968.1	Clostridioides difficile ATCC 9689 = DSM 1296																				
CP003939.1	Clostridioides difficile BJ08																				
NZ_CP026594.1	Clostridioides difficile strain 08-00495																				
NZ_CP010888.1	Clostridioides difficile strain 08ACD0030																				
NZ_CP026599.1	Clostridioides difficile strain 09-00072																				
NZ_CP026596.1	Clostridioides difficile strain 10-00071																				
NZ_CP026597.1	Clostridioides difficile strain 10-00078																				
NZ_CP026598.1	Clostridioides difficile strain 10-00253																				
NZ_CP026593.1	Clostridioides difficile strain 12-00008																				
NZ_CP026595.1	Clostridioides difficile strain 12-00011																				
NZ_CP016318.1	Clostridioides difficile strain 630 delta erm				100	100															
NZ_CP019870.1	Clostridioides difficile strain BR81																				
NZ_CP026592.1	Clostridioides difficile strain CD-10-00484																				
NZ_CP029154.1	Clostridioides difficile strain CD161				99	99															
NZ_CP026591.1	Clostridioides difficile strain CD-17-01474																				
NZ_CP029152.1	Clostridioides difficile strain CDT4																				
NZ_CP022524.1	Clostridioides difficile strain DH/NAP11/106/ST-42																				
NZ_CP020378.1	Clostridioides difficile strain DSM 102859																				
NZ_CP020379.1	Clostridioides difficile strain DSM 102860																				
NZ_CP020380.1	Clostridioides difficile strain DSM 102978																				
NZ_CP011846.1	Clostridioides difficile strain DSM 27638																				
NZ_CP011847.1	Clostridioides difficile strain DSM 27639																				
NZ_CP011848.1	Clostridioides difficile strain DSM 27640																				
NZ_CP012320.1	Clostridioides difficile strain DSM 28196																				
NZ_CP012321.1	Clostridioides difficile strain DSM 28666																				
NZ_CP012309.1	Clostridioides difficile strain DSM 28668																				
NZ_CP012323.1	Clostridioides difficile strain DSM 28669																				
NZ_CP012312.1	Clostridioides difficile strain DSM 28670																				
NZ_CP012325.1	Clostridioides difficile strain DSM 29020																				
NZ_CP016102.1	Clostridioides difficile strain DSM 29627																				
NZ_CP016104.1	Clostridioides difficile strain DSM 29629																				
NZ_CP019860.1	Clostridioides difficile strain DSM 29632																				
NZ_CP016106.1	Clostridioides difficile strain DSM 29637																				
NZ_CP019858.1	Clostridioides difficile strain DSM 29688																				
NZ_CP019857.1	Clostridioides difficile strain DSM 29745																				
NZ_CP019864.1	Clostridioides difficile strain DSM 29747																				
NZ_CP020424.2	Clostridioides difficile strain FDAARGOS_267																				
CP019469.1	Clostridioides difficile strain LEM1																				
NZ_CP025044.1	Clostridioides difficile strain R0104a																				
NZ_CP026613.2	Clostridioides difficile strain R1																				
NZ_CP026614.2	Clostridioides difficile strain R2																				
NZ_CP026615.2	Clostridioides difficile strain R3																				
NZ_CP025047.1	Clostridioides difficile strain W0003a																				
NZ_CP025046.1	Clostridioides difficile strain W0022a																				
NZ_CP025045.1	Clostridioides difficile strain W0023a																				
CP013196.1	Clostridioides difficile strain Z31																				
NC_009089.1	Clostridium difficile 630																				
FN668941.1	Clostridium difficile BI1																				
NC_013315.1	Clostridium difficile CD196																				
FN665654.1	Clostridium difficile strain 2007855																				
FN665652.1	Clostridium difficile CF5																				
NC_017174.1	Clostridium difficile M120																				
FN668375.1	Clostridium difficile M68																				
FN545816.1	Clostridium difficile R20291																				
