# Supplementary material for: Cyclic Diguanylate Regulates Virulence Factor Genes via Multiple Riboswitches in Clostridium difficile
Source: mSphere. 2018 Oct 24;3(5):e00423-18. doi: 10.1128/mSphere.00423-18 (PMC6200980; doi:10.1128/mSphere.00423-18)
Supplement: TABLE S1 [file sph005182673st1.pdf]

Table S1

Lab Notation	Strain/Plasmid Name	Description	Reference
	Escherichia coli DH5α	F- ϕ80*lacZ*ΔM15 Δ(*lacZYA*-*argF*)U169 *recA1 endA1 hsdR*17(rκ -, mκ+) *phoA supE44 thi*-1 *gyrA96 relA1* λ- *tonA*	Invitrogen [1]
RT275	*Bacillus subtilis* BS49	CU2189::Tn916	[2, 3]
RT694	*C. difficile* 630Δerm	Ribotype 012, erythromycin-sensitive derivative of *C. difficile* 630	[4]
RT762	*C. difficile* 630Δerm pMC123::P*cpr*	630Δerm with control vector	[5]
RT763	*C. difficile* 630Δerm pDccA	Strain contains plasmid with the diguanylate cyclase gene *dccA* under the control of the nisin-inducible *cpr* promoter	[5]
RT764	*C. difficile* 630Δerm pDccA^{mut}	Strain contains plasmid with dccA allele encoding enzymatically inactive diguanylate cyclase gene under the control of the nisin-inducible *cpr* promoter	[5]
RT2224	630Δerm pMC-P*tet*	Contains control plasmid	This work
RT2225	630Δerm pCD1987	Expresses putative adhesion under the control of P*tet*	This work
RT2226	630Δerm pCD2795	Expresses putative adhesion under the control of P*tet*	This work
RT2227	630Δerm pCD2796	Expresses putative adhesion under the control of P*tet*	This work
RT2228	630Δerm pCD2797	Expresses putative adhesion under the control of P*tet*	This work
RT2229	630Δerm pCD2831	Expresses putative adhesion under the control of P*tet*	This work
RT2230	630Δerm pCD3246	Expresses putative adhesion under the control of P*tet*	This work
RT2136	630Δerm pSMB47::P_{19900}-*phoZ*	CD630_19900 promoter fused to *phoZ* and integrated on the *C. difficile* chromosome	This work
RT2137	630Δerm pSMB47::P_{19900}-*phoZ* pMC-Pcpr	RT2136 bearing control vector pMC-Pcpr	This work
RT2138	630Δerm pSMB47::P_{19900}-*phoZ* pDccA	RT2136 bearing pDccA	This work
RT2139	630Δerm pSMB47::P_{27970}-*phoZ*	CD630_27970 promoter fused to *phoZ* and integrated on the *C. difficile* chromosome	This work
RT2140	630Δerm pSMB47::P_{27970}-*phoZ* pMC-Pcpr	RT2139 bearing control vector pMC-Pcpr	This work
RT2141	630Δerm pSMB47::P_{27970}-*phoZ* pDccA	RT2139 bearing pDccA	This work
RT2142	630Δerm pSMB47::P_{flgB}-*phoZ*	*flgB* promoter fused to *phoZ* and integrated on the *C. difficile* chromosome	This work
RT2143	630Δerm pSMB47::P_{flgB}-*phoZ* pMC-Pcpr	RT2142 bearing control vector pMC-Pcpr	This work
RT2144	630Δerm pSMB47::P_{flgB}-*phoZ* pDccA	RT2142 bearing pDccA	This work
RT2145	630Δerm pSMB47::P_{19903}-*phoZ*	CD630_19903 promoter fused to *phoZ* and integrated on the *C. difficile* chromosome	This work
RT2146	630Δerm pSMB47::P_{19903}-*phoZ* pMC-Pcpr	RT2145 bearing control vector pMC-Pcpr	This work
RT2147	630Δerm pSMB47::P_{19903}-*phoZ* pDccA	RT2145 bearing pDccA	This work
RT2148	630Δerm pSMB47::P_{23090}-*phoZ*	CD630_23090 promoter fused to *phoZ* and integrated on the *C. difficile* chromosome	This work
RT2149	630Δerm pSMB47::P_{23090}-*phoZ* pMC-Pcpr	RT2148 bearing control vector pMC-Pcpr	This work
RT2150	630Δerm pSMB47::P_{23090}-*phoZ* pDccA	RT2148 bearing pDccA	This work
RT2151	630Δerm pSMB47::P_{33682}-*phoZ*	CD630_33682 promoter fused to *phoZ* and integrated on the *C. difficile* chromosome	This work

RT2152	630Δerm pSMB47::P$_{33682}$-*phoZ* pMC-Pcpr	RT2151 bearing control vector pMC-Pcpr	This work
RT2153	630Δerm pSMB47::P$_{33682}$-*phoZ* pDccA	RT2151 bearing pDccA	This work
RT2154	630Δerm pSMB47::P$_{zmpl}$-*phoZ*	*zmpI* promoter fused to *phoZ* and integrated on the *C. difficile* chromosome	This work
RT2155	630Δerm pSMB47::P$_{zmpl}$-*phoZ* pMC-Pcpr	RT2154 bearing control vector pMC-Pcpr	This work
RT2156	630Δerm pSMB47::P$_{zmpl}$-*phoZ* pDccA	RT2154 bearing pDccA	This work
RT2157	630Δerm pSMB47::P$_{32460}$-*phoZ*	CD630_32460 promoter fused to *phoZ* and integrated on the *C. difficile* chromosome	This work
RT2158	630Δerm pSMB47::P$_{32460}$-*phoZ* pMC-Pcpr	RT2157 bearing control vector pMC-Pcpr	This work
RT2159	630Δerm pSMB47::P$_{32460}$-*phoZ* pDccA	RT2157 bearing pDccA	This work
RT2160	630Δerm pSMB47::P$_{32670}$-*phoZ*	CD630_32670 promoter fused to *phoZ* and integrated on the *C. difficile* chromosome	This work
RT2161	630Δerm pSMB47::P$_{32670}$-*phoZ* pMC-Pcpr	RT2160 bearing control vector pMC-Pcpr	This work
RT2162	630Δerm pSMB47::P$_{32670}$-*phoZ* pDccA	RT2160 bearing pDccA	This work
RT2163	630Δerm pSMB47::P$_{28310}$-*phoZ*	CD630_28310 promoter fused to *phoZ* and integrated on the *C. difficile* chromosome	This work
RT2164	630Δerm pSMB47::P$_{28310}$-*phoZ* pMC-Pcpr	RT2163 bearing control vector pMC-Pcpr	This work
RT2165	630Δerm pSMB47::P$_{28310}$-*phoZ* pDccA	RT2163 bearing pDccA	This work
RT2166	630Δerm pSMB47::P$_{pilA1}$-*phoZ*	*pilA1* promoter fused to *phoZ* and integrated on the *C. difficile* chromosome	This work
RT2167	630Δerm pSMB47::P$_{pilA1}$-*phoZ* pMC-Pcpr	RT2166 bearing control vector pMC-Pcpr	This work
RT2168	630Δerm pSMB47::P$_{pilA1}$-*phoZ* pDccA	RT2166 bearing pDccA	This work
RT2169	630Δerm pSMB47::P$_{pilA1}$-UTR-*phoZ*	*pilA1* promoter and native riboswitch fused to *phoZ* and integrated on the *C. difficile* chromosome	This work
RT2170	630Δerm pSMB47::P$_{pilA1}$-UTR-*phoZ*	RT2169 bearing control vector pMC-Pcpr	This work
RT2171	630Δerm pSMB47::P$_{pilA1}$-UTR-*phoZ*	RT2169 bearing pDccA	This work
RT2172	630Δerm pSMB47::P$_{pilA1}$-UTR^{A70G}-*phoZ*	*pilA1* promoter with A70G mutation in the riboswitch fused to *phoZ* and integrated on the *C. difficile* chromosome	This work
RT2173	630Δerm pSMB47::P$_{pilA1}$-UTR^{A70G}-*phoZ*	RT2172 bearing control vector pMC-Pcpr	This work
RT2174	630Δerm pSMB47::P$_{pilA1}$-UTR^{A70G}-*phoZ*	RT2172 bearing pDccA	This work
	**Plasmid Name**	**Description**	**Reference**
RT264	pMC123	*E. coli – C. difficile* shuttle vector; Amp^R, Cm^R/Tm^R	[6]
RT399	pMC-P$_{cpr}$	pMC123 with *cpr* promoter in the multiple cloning site	[5]
RT402	pDccA	pMC-P$_{cpr}$ ::*dccA* (CD630_14200), encodes DGC	[5]
RT529	pDccA^{mut}	pMC-P$_{cpr}$ ::*dccA*^{mut} (AADEF), encodes inactive DGC	[5]
RT1648	pMC-P$_{tet}$	pMC123 with *tet* promoter	This work

RT1649	pCD1987	pMC-P_{tet}::CD630_19870, putative adhesion under the control of the *tet* promoter	This work
RT1650	pCD2795	pMC-P_{tet}::CD630_27950, putative adhesion under the control of the *tet* promoter	This work
RT1651	pCD2796	pMC-P_{tet}::CD630_27960, putative adhesion under the control of the *tet* promoter	This work
RT1652	pCD2797	pMC-P_{tet}::CD630_27970, putative adhesion under the control of the *tet* promoter	This work
RT1653	pCD2831	pMC-P_{tet}::CD630_28310, putative adhesion under the control of the *tet* promoter	This work
RT1654	pCD3246	pMC-P_{tet}::CD630_32460, putative adhesion under the control of the *tet* promoter	This work
RT2060	pSMB47::P_{19900}::*phoZ*	CD630_19900 putative promoter fused to *phoZ*	This work
RT2061	pSMB47::P_{27970}::*phoZ*	CD630_19900 putative promoter fused to *phoZ*	This work
RT2062	pSMB47::P_{flgB}::*phoZ*	CD630_02450 *flgB* promoter region fused to *phoZ*	This work
RT2063	pSMB47::P_{19903}::*phoZ*	CD630_19903 putative promoter fused to *phoZ*	This work
RT2064	pSMB47::P_{23090}::*phoZ*	CD630_23090 putative promoter fused to *phoZ*	This work
RT2065	pSMB47::P_{33682}::*phoZ*	CD630_33682 putative promoter fused to *phoZ*	This work
RT2066	pSMB47::P_{zmpI}::*phoZ*	CD630_23800 putative promoter fused to *phoZ*	This work
RT2067	pSMB47::P_{32460}::*phoZ*	CD630_32460 putative promoter fused to *phoZ*	This work
RT2068	pSMB47::P_{32670}::*phoZ*	CD630_32670 putative promoter fused to *phoZ*	This work
RT2069	pSMB47::P_{23810}::*phoZ*	CD630_23810 putative promoter fused to *phoZ*	This work
RT2070	pSMB47::P_{pilA1}::*phoZ*	CD630_35130 *pilA1* promoter region fused to *phoZ*	This work
RT2114	pSMB47::P_{pilA1}-UTR-*phoZ*	*pilA1* promoter and native UTR containing the wildtype riboswitch sequence fused to *phoZ*	This work
RT2115	pSMB47::P_{pilA1}-UTR^{A70G}-*phoZ*	*pilA1* promoter and UTR with A70G mutation in the riboswitch sequence fused to *phoZ*	This work

References

1. Hanahan, D., Studies on transformation of *Escherichia coli* with plasmids. J Mol Biol, 1983. **166**(4): p. 557-80.
2. Christie, P.J., et al., Two conjugation systems associated with *Streptococcus faecalis* plasmid pCF10: identification of a conjugative transposon that transfers between *S. faecalis* and *Bacillus subtilis*. J Bacteriol, 1987. **169**(6): p. 2529-36.
3. Browne, H.P., et al., Complete genome sequence of BS49 and draft genome sequence of BS34A, *Bacillus subtilis* strains carrying Tn916. FEMS Microbiol Lett, 2015. **362**(3): p. 1-4.
4. Hussain, H.A., A.P. Roberts, and P. Mullany, Generation of an erythromycin-sensitive derivative of *Clostridium difficile* strain 630 (630Deltaerm) and demonstration that the conjugative transposon Tn916DeltaE enters the genome of this strain at multiple sites. J Med Microbiol, 2005. **54**(Pt 2): p. 137-141.
5. Purcell, E.B., et al., Cyclic diguanylate inversely regulates motility and aggregation in *Clostridium difficile*. J Bacteriol, 2012. **194**(13): p. 3307-3316.
6. McBride, S.M. and A.L. Sonenshein, Identification of a genetic locus responsible for antimicrobial peptide resistance in *Clostridium difficile*. Infect Immun, 2011. **79**(1): p. 167-176.
