# Supplementary material for: Cyclic Diguanylate Regulates Virulence Factor Genes via Multiple Riboswitches in Clostridium difficile
Source: mSphere. 2018 Oct 24;3(5):e00423-18. doi: 10.1128/mSphere.00423-18 (PMC6200980; doi:10.1128/mSphere.00423-18)
Supplement: TABLE S2 [file sph005182673st2.pdf]

Table S2

Lab Notation	Primer name	Sequence (5' to 3')[a]
R1931	Atc_F	TTAGAATTCCATAAAAATAAGAAGCCTGCATTTG
R1932	Atc_R	TTAGAGCTCAGATCTGTTAACGC
R1933	CD630_19870_F	TTAGAGCTCGTAAAGGAGAAAATTTTATGAAATTCTATAAAAGAATATTAACATTGAC
R1934	CD630_19870_R	TATGGATCCACAATTACATCTATTCATTTTCAATTAAATAAC
R1935	CD630_27950_F	AATGGATCCGTAAAGGAGAAAATTTTATGAACAAAAAAATATTATCATTAGGTCTAG
R1936	CD630_27950_R	TATCTGCAGTTTATGGAATTTTTTACAAGCTACC
R1937	CD630_27960_F	AATGAGCTCGTAAAGGAGAAAATTTTATGAATAAAAGAAAATCTTTTATAAGAACTATAG
R1938	CD630_27960_R	TATGGATCCATTTATTTTTCAATTAAACTCTTAATCTTGTC
R1939	CD630_27970_F	AATGAGCTCGTAAAGGAGAAAATTTTATGAAAAAGGCAATATCTTGTGTAC
R1940	CD630_27970_R	TATGGATCCACCTACTATTTATCTCTAACAATATTACC
R1941	CD630_28310_F	AATGAGCTCGTAAAGGAGAAAATTTTATGAAGAAAGGAAATAGAAAGGC
R1942	CD630_28310_R	TATGGATCCTAATGTTAGGGTCTAATTTGTATTTTTATTTC
R1943	CD630_32460_F	AATGAGCTCGTAAAGGAGAAAATTTTTTGAAAACAAAAATTAAAAAATCAAGTATAATC
R1944	CD630_32460_R	TATGGATCCAGATTAATTTCTTTTTATTTTACATTGATACG
R2482	Cdi1-1promF	AGGCTCTCAAGGGCATCGGTCGACTGTTATATATTGTAAAATGTTGAAAAATAGCC
R2483	Cdi1-1promR	ATTTCCTTGTTTCCTCCTGCATGCTTTGCCTATATCTGAATAATAACATAATTTGAAC
R2484	Cdi1-2promF	AGGCTCTCAAGGGCATCGGTCGACAAAATATACACAAAATTATGCTTGTAAGG
R2485	Cdi1-2promR	ATTTCCTTGTTTCCTCCTGCATGCTGCCATTTAATTTTCACTACTTTCTTAC
R2486	Cdi1-3promF	AGGCTCTCAAGGGCATCGGTCGACGTAAAAGACAAATTTAGAATAAATTTATTTTATATTG
R2487	Cdi1-3promR	ATTTCCTTGTTTCCTCCTGCATGCATTTGCCTATTTAGTTTTAACTTAAGTATAC
R2488	Cdi1-8promF	AGGCTCTCAAGGGCATCGGTCGACTCTGGTAATTTTTTATTTAGCATAATATATTCC
R2489	Cdi1-8promR	ATTTCCTTGTTTCCTCCTGCATGCTGCCGATTTAGTTTTATTCTACAAC
R2490	Cdi1-9promF	AGGCTCTCAAGGGCATCGGTCGACTTTTGCATTATTAAAAATGTGTTTTAAATACAAAC
R2491	Cdi1-9promR	ATTTCCTTGTTTCCTCCTGCATGCAGTTTTGCCGATTTAGTTTTATTCTAC
R2492	Cdi1-11promF	AGGCTCTCAAGGGCATCGGTCGACTCAATTTATCTCACTTAAATTAATAACAACTG
R2493	Cdi1-11promR	ATTTCCTTGTTTCCTCCTGCATGCTGCCGATTTAGTTTTATTCTACAATTAC
R2494	Cdi1-12promF	AGGCTCTCAAGGGCATCGGTCGACAAGAAGAAATAAAAATACAAATTAGACCC
R2495	Cdi1-12promR	ATTTCCTTGTTTCCTCCTGCATGCCCTAGTTTTGCCTCATTTAATTTTATATC
R2496	Cdi2-1promF	AGGCTCTCAAGGGCATCGGTCGACATAATGATACTCAAGTATGTGGTTTG
R2497	Cdi2-1promR	ATTTCCTTGTTTCCTCCTGCATGCACAGTTTCTATAAATAGGTTTTTTAATTTTTC
R2498	Cdi2-2promF	AGGCTCTCAAGGGCATCGGTCGACACAAGAGTTATAATATAAAGTAAACATATAATACC
R2499	Cdi2-2promR	ATTTCCTTGTTTCCTCCTGCATGCTAAAAATCTTTTATATATTTTCATAAATTTCTTTTTTATTC
R2500	Cdi2-3promF	AGGCTCTCAAGGGCATCGGTCGACATATAATTGTGTAATTGTTGTAGATAAGG
R2501	Cdi2-3promR	ATTTCCTTGTTTCCTCCTGCATGCTCTATAATATTTCATAAAAACAATTAAAATTGC
R2502	Cdi2-4promF	AGGCTCTCAAGGGCATCGGTCGACTTTCAAATAAATATTAGCAAAGGATGG
R2503	Cdi2-4promR	ATTTCCTTGTTTCCTCCTGCATGCAACACTGTTTATACTATTTATATATACCAAC
R2504	CD630_19900qF	ACTACAATCCCTGATGG
R2505	CD630_19900qR	ACTTAAATGCTCTTCTATGTA
R2506	CD630_19903qF	GCTTATATTAGCAGTTCGT
R2507	CD630_19903qR	CAATACCATTACTTGGTGTA
R2508	CD630_23090qF	ACTGCTTATATTGGTAGTTC
R2509	CD630_23090qR	TGATGCAAGCAAGATTC
R2510	CD630_33682qR[b]	CTTGATGCAAACAAGATTC
R2511	CD630_28300qF	GATGATGTTCCAGGTTTAG
R2512	CD630_28300qR	CTATTGCATGTGCTGTT
R2513	CD630_32460qF	ATCAGATGGTCAGATACC
R2514	CD630_32460qR	TATCTGATGTCTTCTCTCC
R2515	CD630_32670qF	CCTGATATTGGAGATCAAC
R2516	CD630_32670qR	CCCATACGGTTTGAATG
R856	CD0245qF (*flgB*)	GCAACTAATCTAAGAAGTCAGACAATAGC
R857	CD0245qR (*flgB*)	AGGCATAGCATCATTTAGTGTTTCTTC

R930	CD3513qF	TGGCAGTTCCAGCTTTATTTAGTAAT
R931	CD3513qR	AAGATAATGCTGCACTCTTAACTGAA
R850	rpoCqF	CTAGCTGCTCCTATGTCTCACATC
R851	rpoCqR	CCAGTCTCTCCTGGATCAACTA

[a] Restriction sites used for cloning are underlined

[b] Use with RT2507 as the forward primer
