# Supplementary material for: Cyclic Diguanylate Regulates Virulence Factor Genes via Multiple Riboswitches in Clostridium difficile
Source: mSphere. 2018 Oct 24;3(5):e00423-18. doi: 10.1128/mSphere.00423-18 (PMC6200980; doi:10.1128/mSphere.00423-18)
Supplement: TABLE S3 [file sph005182673st3.pdf]

Table S3

Locus Tag Identifier	Gene Name	Gene Product	Fold Change (pDccA/vector) [a]	Riley Functional Class [b]
CD630_01300	*metK*	S-adenosylmethionine synthetase	-2.15	3.3.16
CD630_01480		uncharacterised protein	-4.40	7.0.0
CD630_01740	*cooS*	Carbon monoxide dehydrogenase	-8.61	3.5.2
CD630_01750		putative oxidoreductase, Fe-S subunit	-5.15	3.5.3
CD630_01910		putative DNA glycosylase	-2.06	2.2.3
CD630_02110	*licC*	CTP:phosphocholine cytidylyltransferase	-10.96	4.1.9
CD630_02120		putative sulfatase	-9.03	3.3.19
CD630_02130		putative spore coat protein	-4.79	1.8.1
CD630_02140		uncharacterised protein	-5.77	7.0.0
CD630_02260		putative lytic transglycosylase	-6.05	3.3.21
CD630_02270		conserved hypothetical protein	-5.77	0.0.2
CD630_02290	*flgM*	Negative regulator of flagellin synthesis (Anti-sigma-d factor)	-5.49	6.2.2
CD630_02300		putative flagellar biosynthesis protein	-5.56	1.1.1
CD630_02310	*flgK*	Flagellar hook-associated protein FlgK (or HAP1)	-5.53	1.1.1
CD630_02320	*flgL*	Flagellar hook-associated protein FlgL (or HAP3)	-5.68	1.1.1
CD630_02330	*fliW*	Flagellar assembly factor FliW	-5.93	1.1.1
CD630_02340	*csrA*	Carbon storage regulator CsrA	-5.99	6.5.0
CD630_02350	*fliS1*	Flagellar protein FliS1	-6.01	1.1.1
CD630_02360	*fliS2*	Flagellar protein FliS2	-6.03	1.1.1
CD630_02370	*fliD*	Flagellar hook-associated protein 2 FliD (or HAP2)	-6.45	1.1.1
CD630_02380		conserved hypothetical protein	-6.76	0.0.2
CD630_02390	*fliC*	Flagellin C	-7.85	1.1.1
CD630_02400		Glycosyltransferase	-5.71	2.2.4
CD630_02410		flagella glycosylation phosphoserine phosphatase	-5.88	2.2.4
CD630_02420		putative nucleoside triphosphate transferase	-5.92	2.2.4
CD630_02430		flagella glycosylation methyltransferase domain protein	-5.96	2.2.4
CD630_02440		putative CDP-glycerol:Poly(glycerophosphate) glycerophosphotransferase	-5.89	2.2.4
CD630_02450	*flgB*	Flagellar basal-body rod protein FlgB	-15.09	1.1.1
CD630_02460	*flgC*	Flagellar basal-body rod protein FlgC	-14.93	1.1.1
CD630_02470	*fliE*	Flagellar hook-basal body complex protein FliE	-15.47	1.1.1
CD630_02480	*fliF*	Flagellar M-ring protein FliF	-13.28	1.1.1
CD630_02490	*fliG*	Flagellar motor switch protein FliG	-12.07	1.1.1
CD630_02500	*fliH*	Flagellar assembly protein FliH	-11.00	1.1.1
CD630_02510	*fliI*	ATP synthase subunit beta FliI	-11.23	1.1.1
CD630_02520	*fliJ*	Flagellar protein FliJ	-11.73	1.1.1
CD630_02530	*fliK*	Flagellar hook-length control protein FliK	-11.30	1.1.1
CD630_02540	*flgD*	Basal-body rod modification protein FlgD	-11.76	1.1.1
CD630_02550	*flgE*	Flagellar hook protein FlgE (Distal rod protein)	-11.68	1.1.1
CD630_02551	*FlbD*	Flagellar protein FlbD	-12.10	1.1.1
CD630_02560	*motA*	Flagellar motor rotation protein MotA	-11.53	1.1.1
CD630_02570	*motB*	Flagellar motor rotation protein MotB (Chemotaxis protein MotB)	-11.45	1.1.1
CD630_02580	*fliL*	Flagellar basal body-associated protein FliL	-11.51	1.1.1
CD630_02590	*fliZ*	Flagellar protein FliZ	-11.37	1.1.1
CD630_02600	*fliP*	Flagellar biosynthesis protein FliP	-10.76	1.1.1
CD630_02610	*fliQ*	Flagellar biosynthetic protein FliQ	-11.10	1.1.1
CD630_02620	*flhB*	Bifunctional flagellar biosynthesis protein FliR/FlhB	-10.25	1.1.1
CD630_02630	*flhA*	Flagellar biosynthesis protein FlhA	-10.14	1.1.1

CD630_02640	*flhF*	Flagellar biosynthesis regulator FlhF (Flagella-associated GTP-binding protein)	-9.89	1.1.1
CD630_02650	*flhG*	Flagellar number regulator FlhG	-9.76	1.1.1
CD630_02660	*fliA*	RNA polymerase sigma-28factor for flagellar operon	-9.94	6.2.1
CD630_02670		putative flagellar protein	-10.10	1.1.1
CD630_02671		putative flagellar protein	-11.82	1.1.1
CD630_02680	*flgG1*	Flagellar hook-basal body complex protein FlgG1	-9.59	1.1.1
CD630_02690	*flgG*	Flagellar basal body rod protein FlgG	-10.09	1.1.1
CD630_02700	*fliM*	Flagellar motor switch protein FliM	-9.66	1.1.1
CD630_02710	*fliN1*	Flagellar motor switch phosphatase FliN1	-5.85	1.1.1
CD630_02280	*fliN*	Flagellar motor switch protein FliN	-10.19	1.1.1
CD630_02720		conserved hypothetical protein	-9.93	0.0.2
CD630_02730	*htpG*	Heat shock protein 90 (Heat shock protein HtpG)(High temperature protein G)	-4.17	1.3.1
CD630_02731		conserved hypothetical protein	-3.05	0.0.2
CD630_02850		PTS system, mannose/fructose/sorbose IIB component	2.75	1.5.3
CD630_02860		PTS system, mannose/fructose/sorbose IIA component	2.43	1.5.3
CD630_02880		PTS system, mannose/fructose/sorbose IIC component	2.84	1.5.3
CD630_05110	*tndX*	Recombinase site-specific resolvase family Tn5397, CTn3-Orf3	-12.90	5.1.4
CD630_05290		putative membrane protein	-5.06	4.1.6
CD630_05600	*nfo*	Endonuclease IV	-5.33	2.2.3
CD630_05800	*gapN*	Glyceraldehyde-3-phosphate dehydrogenase (NADP(+)) (GADPH)	-11.79	3.5.5
CD630_05870		uncharacterised protein	2.02	7.0.0
CD630_05880		uncharacterised protein	2.06	7.0.0
CD630_06180		Transcriptional regulator, LytR family	-2.59	6.5.0
CD630_06190		conserved hypothetical protein	-7.92	0.0.2
CD630_06200		conserved hypothetical protein	-8.54	0.0.2
CD630_06210		putative membrane protein	-6.87	4.1.6
CD630_06220		conserved hypothetical protein	-7.09	0.0.2
CD630_06720		conserved hypothetical protein	-4.36	0.0.2
CD630_07570		putative c-di-GMP phosphodiesterase	-5.35	6.5.0
CD630_10910	*int1*	Integrase Tn1549-like, CTn4-Orf34	2.68	5.1.4
CD630_11030		putative conjugative transposon protein Tn1549-like, CTn4-Orf21	4.91	5.1.4
CD630_11031		putative conjugative transposon protein Tn1549-like, CTn4-Orf20	3.90	5.1.4
CD630_11040		putative conjugative transposon protein Tn1549-like, CTn4-Orf19	3.03	5.1.4
CD630_11250		Nitroreductase-family protein	-6.05	7.0.0
CD630_11260		Transcriptional regulator, AraC family	-4.61	6.3.2
CD630_12790	*iscS2*	Cysteine desulfurase	-5.22	3.3.19
CD630_12800		fe-s iron-sulfur cluster assembly protein, nifu family	-3.54	3.5.3
CD630_14700		putative rhodanese-like domain-containing protein	-19.49	1.4.2
CD630_14740		putative rubrerythrin (Rr)	-8.36	1.4.2
CD630_14790	*feoB1*	Ferrous iron transport protein B	2.24	1.6.3
CD630_15240		putative rubrerythrin	-2.56	1.4.2
CD630_15680		conserved hypothetical protein	-7.98	0.0.2
CD630_15800	*hom2*	Homoserine dehydrogenase	2.73	3.1.0
CD630_16970	*ribH*	6,7-dimethyl-8-ribityllumazine synthase	-3.74	3.2.13
CD630_16980	*ribBA*	Riboflavin biosynthesis protein ribBA [Includes: 3,4-dihydroxy-2-butanone 4-phosphate synthase; GTP cyclohydrolase-2]	-2.74	3.2.13

CD630_17290		putative sodium:phosphate symporter	-9.01	1.5.2
CD630_17770		putative arsenate reductase	-5.61	1.4.2
CD630_17940		conserved hypothetical protein	5.11	0.0.2
CD630_17950		conserved hypothetical protein	5.76	0.0.2
CD630_18220	*bcp*	putative thiol peroxidase	-13.15	1.4.2
CD630_18230		conserved hypothetical protein, UPF0246 family	-10.41	0.0.2
CD630_18240		P-type calcium transport ATPase	-3.15	1.5.2
CD630_18820		Site-specific recombinase	-3.84	5.1.4
CD630_18990		putative dCMP deaminase	-13.88	7.0.0
CD630_19870	*cwp28*	putative cell wall binding protein cwp28	4.31	4.1.8
CD630_19900		putative protein with SH3 domain	35.08	0.0.2
CD630_19903		conserved hypothetical protein	-2.00	0.0.2
CD630_20460		conserved hypothetical protein	-9.50	0.0.2
CD630_21151		uncharacterised protein	-11.51	7.0.0
CD630_21170	*trxB2*	Thioredoxin reductase	-5.23	3.2.15
CD630_21580	*gabT*	4-aminobutyrate aminotransferase	2.06	3.3.20
CD630_22090		putative GTP-binding protein, HflX type	-4.09	7.0.0
CD630_23090		conserved hypothetical protein	-2.64	0.0.2
CD630_23270	*gatA*	PTS system, fructose/mannitol family IIA component	5.32	1.5.3
CD630_24760		gnat family acetyltransferase	-3.38	7.0.0
CD630_24770		uncharacterised protein	-4.50	7.0.0
CD630_24860		PTS system, fructose-like IIC component	-2.44	1.5.3
CD630_24870		PTS system, fructose-like IIB component	-2.64	1.5.3
CD630_24880		PTS system, fructose-like IIA component	-3.23	1.5.3
CD630_24890		Transcription antiterminator, PTS operon regulator	-3.91	6.5.0
CD630_27950	*selA*	L-seryl-tRNA(Sec) selenium transferase (Selenocysteine synthase) (Sec synthase) (Selenocysteinyl-tRNA (Sec) synthase)	2.89	2.2.1
CD630_27960	*selD*	Selenide, water dikinase (Selenophosphate synthetase) (Selenium donor protein)	-4.07	2.2.1
CD630_27970	*comE*	Competence protein ComEA	-7.14	4.1.6
CD630_28300		uncharacterised protein	-65.96	7.0.0
CD630_28310		putative adhesin	42.42	4.1.5
CD630_28410		putative amidohydrolase	-11.37	3.4.5
CD630_28450	*rbr1*	Rubrerythrin	2.50	1.4.2
CD630_28460		conserved hypothetical protein	2.42	0.0.2
CD630_28470		conserved hypothetical protein	2.38	0.0.2
CD630_28480		conserved hypothetical protein	2.37	0.0.2
CD630_28750	*fhuC*	ABC-type transport system, ferrichrome-specific ATP-binding protein	-3.78	1.6.3
CD630_29950	*nrdE*	Ribonucleoside-diphosphate reductase subunit alpha (Ribonucleotide reductase large subunit)	-4.39	3.3.11
CD630_30100		putative cytochrome C assembly protein	-2.30	3.2.6
CD630_30330	*trxA1*	Thioredoxin	-3.78	3.2.15
CD630_30390		putative ATPase	-6.98	7.0.0
CD630_30400		conserved hypothetical protein	-10.44	0.0.2
CD630_30990		putative amidohydrolase, M20D family	-2.08	2.1.4
CD630_31000		putative C4-dicarboxylate anaerobic carrier,DcuC family	-2.09	1.5.3
CD630_32110		conserved hypothetical protein	2.07	0.0.2
CD630_32150		ABC-type transport system, glycine betaine/carnitine/choline ATP-binding protein	-5.74	1.6.2
CD630_32160		ABC-type transport system, glycine betaine/carnitine/choline permease	-8.39	1.6.2
CD630_32460		putative surface protein	4.28	4.1.8

CD630_32640		Fragment of conserved hypothetical protein (C-terminal region)	3.37	0.0.2
CD630_32650		Two-component response regulator	17.57	6.1.2
CD630_32660		Two-component sensor histidine kinase	18.37	6.1.1
CD630_32670		Two-component response regulator	18.74	6.1.2
CD630_33230		conserved hypothetical protein	-2.25	0.0.2
CD630_33250		conserved hypothetical protein	2.10	0.0.2
CD630_33260		Integrase, lambdoid phage family Tn916-like,CTn6-Orf1	2.14	5.1.2
CD630_33270		putative conjugative transposon protein Tn916-like, CTn6-Orf2	2.12	5.1.4
CD630_34770		putative dCMP deaminase	-2.00	3.3.11
CD630_35040		putative type IV prepilin peptidase, A24A family	3.18	4.1.5
CD630_35050		putative twitching motility protein PilT	3.49	4.1.5
CD630_35060		conserved hypothetical protein	4.28	0.0.2
CD630_35070		putative type IV pilin	4.40	4.1.5
CD630_35080		putative type IV pilin	4.69	4.1.5
CD630_35090		putative type IV pilus assembly protein	4.67	4.1.5
CD630_35100		putative membrane protein	5.08	4.1.6
CD630_35110		putative type IV pilus secretion protein	5.28	4.1.5
CD630_35120		putative type IV pilus transporter system, ATP-binding	4.89	4.1.5
CD630_35130		putative pilin protein PilA1	11.72	4.1.5
CD630_35250		ABC-type transport system, iron-family extracellular solute-binding protein	-9.35	1.5.5
CD630_35260		ABC-type transport system, iron-family permease	-9.01	1.5.5
CD630_35270		ABC-type transport system, iron-family ATP-binding protein	-9.81	1.5.5
CD630_36100		putative glyoxalase	-11.82	1.4.2
CD630_36400		putative ribokinase family sugar kinase	-2.01	3.4.3
CD630_36410		conserved hypothetical protein	-4.38	0.0.2
CD630_36420		putative selenocysteine synthase	-4.98	2.2.1
CD630_36430		Dihydroorotase	-5.02	3.7.2

[a] Shown are genes with at least 2-fold differential expression between *C. difficile* with vector and pDccA and meeting p<0.05 after Bonferroni's correction.

[b] Riley Class identification based on Pettit et al. BMC Genomics 2014.
